# In-hospital mortality risk stratification of Asian ACS patients with artificial intelligence algorithm

**DOI:** 10.1371/journal.pone.0278944

**Published:** 2022-12-12

**Authors:** Sazzli Kasim, Sorayya Malek, Cheen Song, Wan Azman Wan Ahmad, Alan Fong, Khairul Shafiq Ibrahim, Muhammad Shahreeza Safiruz, Firdaus Aziz, Jia Hui Hiew, Nurulain Ibrahim

**Affiliations:** 1 Cardiology Department, Faculty of Medicine, Universiti Teknologi MARA (UiTM), Shah Alam, Malaysia; 2 Cardiac Vascular and Lung Research Institute, Universiti Teknologi MARA (UiTM), Shah Alam, Malaysia; 3 National Heart Association of Malaysia, Heart House, Kuala Lumpur, Malaysia; 4 Faculty of Medicine, Universiti Teknologi MARA (UiTM), Sungai Buloh Campus, Sungai Buloh, Malaysia; 5 Bioinformatics Division, Institute of Biological Sciences, Faculty of Science, University of Malaya, Kuala Lumpur, Malaysia; 6 Division of Cardiology, University Malaya Medical Centre, Kuala Lumpur, Malaysia; 7 Sarawak Heart Centre, Kota Samarahan, Sarawak, Malaysia; 8 Clinical Research Centre, Sarawak General Hospital, Institute for Clinical Research, National Institutes of Health, Jalan Hospital, Kuching, Sarawak, Malaysia; 9 Swinburne University of Technology, Sarawak Campus, Kuching, Malaysia; 10 Department of Artificial Intelligence, Faculty of Computer Science and Information Technology, University of Malaya, Kuala Lumpur, Malaysia; Samsung Medical Center, REPUBLIC OF KOREA

## Abstract

**Background:**

Conventional risk score for predicting in-hospital mortality following Acute Coronary Syndrome (ACS) is not catered for Asian patients and requires different types of scoring algorithms for STEMI and NSTEMI patients.

**Objective:**

To derive a single algorithm using deep learning and machine learning for the prediction and identification of factors associated with in-hospital mortality in Asian patients with ACS and to compare performance to a conventional risk score.

**Methods:**

The Malaysian National Cardiovascular Disease Database (NCVD) registry, is a multi-ethnic, heterogeneous database spanning from 2006–2017. It was used for in-hospital mortality model development with 54 variables considered for patients with STEMI and Non-STEMI (NSTEMI). Mortality prediction was analyzed using feature selection methods with machine learning algorithms. Deep learning algorithm using features selected from machine learning was compared to Thrombolysis in Myocardial Infarction (TIMI) score.

**Results:**

A total of 68528 patients were included in the analysis. Deep learning models constructed using all features and selected features from machine learning resulted in higher performance than machine learning and TIMI risk score (p < 0.0001 for all). The best model in this study is the combination of features selected from the SVM algorithm with a deep learning classifier. The DL (SVM selected var) algorithm demonstrated the highest predictive performance with the least number of predictors (14 predictors) for in-hospital prediction of STEMI patients (AUC = 0.96, 95% CI: 0.95–0.96). In NSTEMI in-hospital prediction, DL (RF selected var) (AUC = 0.96, 95% CI: 0.95–0.96, reported slightly higher AUC compared to DL (SVM selected var) (AUC = 0.95, 95% CI: 0.94–0.95). There was no significant difference between DL (SVM selected var) algorithm and DL (RF selected var) algorithm (p = 0.5). When compared to the DL (SVM selected var) model, the TIMI score underestimates patients’ risk of mortality. TIMI risk score correctly identified 13.08% of the high-risk patient’s non-survival vs 24.7% for the DL model and 4.65% vs 19.7% of the high-risk patient’s non-survival for NSTEMI. Age, heart rate, Killip class, cardiac catheterization, oral hypoglycemia use and antiarrhythmic agent were found to be common predictors of in-hospital mortality across all ML feature selection models in this study. The final algorithm was converted into an online tool with a database for continuous data archiving for prospective validation.

**Conclusions:**

ACS patients were better classified using a combination of machine learning and deep learning in a multi-ethnic Asian population when compared to TIMI scoring. Machine learning enables the identification of distinct factors in individual Asian populations to improve mortality prediction. Continuous testing and validation will allow for better risk stratification in the future, potentially altering management and outcomes.

## Introduction

Acute coronary syndrome (ACS), also known as a heart attack, is a leading cause of death and disability in the Asian region, with an in-hospital mortality rate of more than 5% [1]. Coronary artery disease (CAD) is responsible for 20–25% of deaths in public hospitals in South-East Asia [2, 3] and frequently manifest as ACS. The three clinical manifestations of ACS are STEMI, non-STEMI, and unstable angina (UA) [4, 5].

In ACS patients, Thrombolysis in Myocardial Infarction (TIMI) and the Global Registry of Acute Coronary Events (GRACE) [6] used in clinical guidelines to predict mortality risk. The TIMI STEMI Risk score is only for STEMI patients; NSTEMI patients require a different TIMI score, NSTEMI. GRACE is applicable in both scenarios. Big data techniques could provide additional insight because TIMI and GRACE only cover traditional prognostic factors [7]. The requirement to wait for blood results measuring renal function restricts the use of the GRACE risk score in practice and delays prediction.

The TIMI and GRACE risk scores were calculated using data from a Western Caucasian cohort with limited participation from an Asian cohort. Asian patients have been understudied [8], and Asians are more likely to develop ACS, diabetes, hypertension, and chronic kidney disease at a younger age and seek medical attention later [8–10]. The bi-annual NCVD ACS Registry, which is publicly available online, publishes evidence of a higher prevalence of risk factors as well as earlier onset of heart disease [11]. Similar findings are seen in registry data from Korea (KAMIR registry), Singapore (SAMIR), and the Gulf countries. This is in reference to well-published data derived primarily from Western literature and utilizing Caucasians in their database [12].

A model that can better predict ACS patient mortality will improve prognosis. A mortality risk scoring system based on machine learning (ML) and deep learning (DL) algorithms reduces information loss from conventional risk scores [13].

These algorithms have been found to be useful in calculating mortality risk in our previous study in patients with STEMI [14–16]. Similar studies in Korean and Chinese only population have also been reported [17–19].

In a population-specific registry, DL and ML algorithms outperformed conventional risk scoring methods like TIMI and GRACE risk score in mortality prediction post-STEMI [20–24] and ACS [7, 25].

Different ML algorithms and features chosen from these ML algorithms on population-specific datasets enables the identification of distinct factors for improved mortality prediction over TIMI [20, 26–28]. Because different algorithms result in different features being selected, it is possible to compare which algorithm and combination of features will produce better results than the TIMI risk score.

When compared to traditional ML algorithms, DL outperformed in terms of in-hospital mortality post ACS, reducing the need for feature engineering and extraction [7, 29, 30]. DL algorithms automatically learn features and classify data better than conventional ML [31, 32]. To improve model performance, ML algorithms require feature selection methods [33]. Unlike ML algorithms, the interpretation of the important variables for the decision of the risk scores is unknown in DL models [7].

Identifying risk factors for mortality improves clinical patient care. To better understand DL’s "black box" feature selection, we incorporate ML features into the DL model as in Kasim’s research [34].We anticipate that integrating DL and ML feature selection algorithms can improve model accuracy and understanding of factors associated with in-hospital mortality prediction in Asian ACS patients. Additionally, we intend to compare the performance of ML with that of DL developed utilising both complete and selected features from the ML feature selection algorithm. We also aim to verify the developed ML and DL prediction models against the TIMI risk score, utilizing multi-ethnic registry data on Asian ACS patients.

## Materials and methods

### Study data

We used data from Malaysian National Cardiovascular Database (NCVD-ACS) registries from 2006 to 2017 from ten participating hospitals. The NCVD registry was approved by the Malaysian Ministry of Health (MOH) in 2007. (Approval Code: NMRR-07-20-250). It waived NCVD informed patient consent and the patient information was anonymize to be use in our study. In addition to outcomes, the registry collects data on a predefined set of clinical, demographic and procedural factors from participants [20, 25, 35]. The UITM ethics committee (Reference number: 600-TNCPI (5/1/6)) and the National Heart Association of Malaysia (NHAM) authorised the study with the approval code REC/673/19. The UiTM Ethics Committee operates in accordance to the ICH Good Clinical Practice Guidelines, Malaysia Good Clinical Practice Guidelines and Declaration of Helsinki.

All patients from the ACS registry without exclusion were used including patients who received reperfusion (fibrinolysis, primary PCI (PPCI), angiography demonstrating spontaneous reperfusion, or urgent coronary artery bypass grafting (CABG)). In this context, STEMI was defined as persistent ST-segment elevation ≥1mm in two contiguous electrocardiographic leads, or the presence of a new left bundle branch block in the setting of positive cardiac markers. NSTEMI is defined by the presence of acute chest pain with positive cardiac markers but without persistent ST-segment elevation [36].

This study examined 54 variables drawn from a comprehensive set of data derived from clinical guidelines. Sociodemographic characteristics, CVD diagnosis and severity, CVD risk factors, CVD comorbidities, non-CVD comorbidities, biomarkers, and medication use were all included in the variables. In-hospital mortality was calculated from the time of initial hospitalization. The Malaysian National Registration Department confirms fatalities on an annual basis. The registry’s data excludes short-term complications such as heart failure. The study discarded follow-up data points due to an excessive number of missing values. To maximize the study’s impact, we focused our algorithm on potentially policy-changing outcomes that is mortality. Several more publications make a similar point [7, 20, 37].

### Classification and sample pre-processing

#### Complete cases

We used a complete set of data to ensure the validity of the findings for model development for the primary analysis. A total of 68,528 ACS cases from the registry were collected and **13,190** were identified as complete cases (with no missing values on predictors). This rendered complete cases of patients with a full predictor set of 54 variables (10 continuous, 44 categorical).

#### Missing cases

Secondary analyses on the best performing algorithm were carried out on the 68,528 ACS cases that includes missing cases. Our imputation dataset model was based on Wallert et al. study [38]. In the study, two different models were developed for training and testing using both complete and imputed cases. Comparing the performance of both models revealed that imputed analyses produced comparable results to the full case model.

We used two imputation approaches from R package missForest [39] and multivariable imputation using predictive mean matching [40].

Our definition of an incomplete dataset includes missing variables of up to 30 percent. There are no missing data for electrocardiography, age, or gender; however, there are fewer than 15% missing data for race (3%), pharmaceutical therapy (2%-14%), invasive therapeutic procedures (less than 8%), clinical representation (less than 3%) and status before ACS occurrence (5%-15%). Up to 30% of the data for baseline intervention variables and Killip class is missing.

The missing dataset referenced is for patient characteristics, not outcomes. Due to the prospective structure of our dataset and the retroactive administration of data, the proportion of missing values across all variables was completely unpredictable and beyond our control. The probability of missing values in our dataset is independent of both the observed values in any variable and the unseen portion of the dataset.

Consequently, the dataset is categorized as missing completely at random (MCAR), which suggests that the pattern of missing values is independent of any variable that may or may not be included in the study. Table 1 shows the baseline characteristics for complete set and imputed dataset.

**Table 1:** Baseline characteristics for complete set and imputed dataset.

Variables		Complete Set (No Data Imputation)				With Data Imputation			
		All (n = 13190)	Alive (n = 12512)	Died (n = 678)	p-value	All (n = 68528)	Alive (n = 63719)	Died (n = 4809)	p-value
**Demographic**									
**Patient Age**		58.42 ± 12.04	58.07 ± 11.97	64.98 ± 11.42	<0.001*	58.64 ± 12.22	58.21± 12.15	64.42 ± 11.74	<0.001*
**Sex**					<0.001*				<0.001*
	1: Male	10515(79.7%)	10018 (80.1%)	497 (73.3%)		53694 (78.4%)	50179 (78.8%)	3515 (73.1%)	
	2: Female	2675 (20.3%)	2494 (19.9%)	181 (26.7%)		14834 (21.6%)	13540 (21.2%)	1294 (26.9%)	
**Race**					0.012				<0.001*
	1: Malay	6724 (51.0%)	6355 (50.8%)	369 (54.4%)		35884 (52.4%)	33139 (52.0%)	2745 (57.1%)	
	2: Chinese	3087 (23.4%)	2915 (23.3%)	172 (25.4%)		14861 (21.7%)	13776 (21.6%)	1085 (22.6%)	
	3: Indian	2703 (20.5%)	2593 (20.7%)	110 (16.2%)		13781 (20.1%)	13015 (20.4%)	766 (15.9%)	
	4. Others	676 (5.1%)	649 (5.2%)	27 (4.0%)		4002 (5.8%)	3789 (5.9%)	213 (4.4%)	
**Status Before Event**									
**Smoking status**					<0.001*				<0.001*
	1: Never	5352 (40.6%)	5027 (40.2%)	325 (47.9%)		27932 (40.8%)	25727 (40.4%)	2205 (45.9%)	
	2: Former	2527(19.2%)	2389 (19.1%)	138 (20.4%)		14810 (21.6%)	13758 (21.6%)	1052 (21.9%)	
	3: Current	5311(40.3%)	5096 (40.7%)	215 (31.7%)		25786 (37.6%)	24234 (38.0%)	1552 (32.3%)	
**Dyslipidemia (cdys)**					0.157				<0.001*
	1: Yes	4791(36.3%)	4562 (36.5%)	229 (33.8%)		29475 (43.0%)	27678 (43.4%)	1797 (37.4%)	
	2: No	8399 (63.7%)	7950 (63.5%)	449 (66.2%)		39053 (57.0%)	36041 (56.6%)	3012 (62.6%)	
**History of Diabetes(cdm)**					<0.001*				<0.001*
	1: Yes	5743(43.5%)	5372 (42.9%)	371 (54.7%)		32074 (46.8%)	29332 (46.0%)	2742 (57.0%)	
	2: No	7447 (56.5%)	7140 (57.1%)	307 (45.3%)		36454 (53.2%)	34387 (54.0%)	2067 (43.0%)	
**History of Hypertension** **(chpt)**					<0.001*				<0.001*
	1: Yes	8247 (62.5%)	7774 (62.1%)	473 (68.8%)		45932 (67.0%)	42511 (66.7%)	3421 (71.1%)	
	2: No	4943 (37.5%)	4738 (37.9%)	205 (30.2%)		22596 (33.0%)	21208 (33.3%)	1388 (28.9%)	
**Family History of Premature Cardiovascular Disease (cpremcvd)**					<0.001*				<0.001*
	1: Yes	1698 (12.9%)	1653 (13.2%)	45 (6.6%)		10856 (15.8%)	10426 (16.4%)	430 (8.9%)	
	2: No	11492 (87.1%)	10859 (86.8%)	633 (93.4%)		57672 (84.2%)	53293 (83.6%)	4379 (91.1%)	
**History of Myocardial Infection (cmi)**					0.009				0.074
	1: Yes	1797 (13.6%)	1682 (13.4%)	115 (17.0%)		13924 (20.3%)	12995 (20.4%)	929 (19.3%)	
	2: No	11393 (86.4%)	10830 (86.6%)	563 (83.0%)		54604 (79.7%)	50724 (79.6%)	3880 (80.7%)	
**Documented CAD (ccap)**					0.385				<0.001*
	1: Yes	2722 (20.6%)	2591 (20.7%)	131 (19.3%)		18053 (26.3%)	17050 (26.8%)	1003 (20.9%)	
	2: No	10468 (79.4%)	9921 (79.3%)	547 (80.7%)		50475 (73.3%)	46669 (73.2%)	3806 (79.1%)	
**Chronic angina > 2week** **(canginamt2wk)**					0.416				<0.001*
	1: Yes	837 (6.3%)	799 (6.4%)	38 (5.6%)		6955 (10.1%)	6555 (10.3%)	400 (8.3%)	
	2: No	12353 (93.7)	11713 (93.6%)	640 (92.4%)		61573 (89.9%)	57164 (89.7%)	4409 (91.7%)	
**New onset angina** **(canginapast2wk)**					0.112				<0.001*
	1: Yes	8535 (64.7%)	8077 (64.6%)	458 (67.6%)		45660 (66.6%)	42589 (66.8%)	3071 (63.9%)	
	2: No	4655 (35.3%)	4435 (35.4%)	220 (32.4%)		22868 (33.4%)	21130 (33.2%)	1738 (36.1%)	
**History of Heart Failure (cheartfail)**					<0.001*				<0.001*
	1: Yes	595 (4.5%)	530 (4.2%)	65 (9.6%)		4797 (7.0%)	4232 (6.6%)	565 (11.7%)	
	2: No	12595 (95.5%)	11982 (95.8%)	613 (90.4%)		63731 (93.0%)	59487 (93.4%)	4244 (88.3%)	
**History of Chronic Lung Disease (clung)**					0.004				<0.001*
	1: Yes	401 (3.0%)	368 (2.9%)	33 (4.9%)		2445 (3.6%)	2183 (3.4%)	262 (5.4%)	
	2: No	12789 (97.0%)	12144 (97.1%)	645 (95.1%)		66083 (96.4%)	61536 (96.6%)	4547 (94.6%)	
**History of Renal Disease (crenal)**					<0.001*				<0.001*
	1: Yes	873 (6.6%)	774 (6.2%)	99 (14.6%)		5757 (8.4%)	5010 (7.9%)	747 (15.5%)	
	2: No	12317 (93.4%)	11738 (93.8%)	579 (85.4%)		62771 (91.6%)	58709 (92.1%)	4062 (84.5%)	
**History of Cerebrovascular Disease (ccerebrovascular)**					<0.001*				<0.001*
	1: Yes	457 (3.5%)	417 (3.3%)	40 (5.9%)		2915 (4.3%)	2590 (4.1%)	325 (6.8%)	
	2: No	12733 (96.5%)	12095 (96.7%)	638 (94.1%)		65613 (95.7%)	61129 (95.9%)	4484 (93.2%)	
**History of Peripheral vascular Disease (cpvascular)**					0.046				<0.001*
	1: Yes	54 (0.4%)	48 (0.4%)	6 (0.9%)		546 (0.8%)	486 (0.8%)	60 (1.2%)	
	2: No	13136 (99.6%)	12464 (99.6%)	672 (99.1%)		67982 (99.2%)	63233 (99.2%)	4749 (98.9%)	
**Clinical Presentation and Examination**									
**Heart rate (bpm)**		83.34 ± 20.66	82.62 ± 20.09	96.60 ± 25.83	<0.001*	83.33 ± 20.89	82.54 ± 20.17	93.92 ± 26.57	<0.001*
**Systolic Blood Pressure (mmHg) (bpsys)**		138.84 ± 27.92	139.61 ± 27.52	124.67 ± 31.30	<0.001*	138.80 ± 28.73	82.52 ± 20.18	122.36 ± 31.93	<0.001*
**Diastolic Blood Pressure (mmHg) (bpdias)**		81.68 ± 17.49	82.03 ± 17.31	75.31 ± 19.47	<0.001*	81.30 ± 17.56	140.04 ± 28.08	73.03±19.88	<0.001*
**Killip Classifications**					<0.001*				<0.001*
	1: Killip 1	9336 (70.8%)	9156 (73.2%)	180 (26.5%)		47131 (68.8%)	45757 (71.8%)	1374 (28.6%)	
	2: Killip 2	2255 (17.1%)	2131 (17.0%)	124 (18.3%)		12132 (17.7%)	11220 (17.6%)	912 (19.0%)	
	3: Killip 3	610 (4.6%)	527 (4.2%)	83 (12.2%)		3341 (4.9%)	2793 (4.4%)	548 (11.4%)	
	4: Killip 4	989 (7.5%)	698 (5.6%)	291 (42.9%)		5924 (8.6%)	3949 (6.2%)	1975 (41.1%)	
**Peak CK**		1323.86 ± 1915.34	1267.64 ± 1803.80	2361.22 ± 3194.35	<0.001*	1954.10 ± 225995.36	1957.21 ± 234356.12	1912.92 ± 8783.43	0.962
**Baseline Investigation**									
**Total Cholesterol (mmol/L)**		5.12 ± 1.37	5.15 ± 1.36	4.64 ± 1.52	<0.001*	5.08 ± 1.43	5.10 ± 1.42	4.73 ± 1.53	<0.001*
**HDL (mmol/L)**		1.09 ± 0.34	1.09 ± 0.33	1.06 ± 0.40	0.022	1.09 ± 0.35	1.92 ± 0.35	1.09 ± 0.37	0.220
**LDL (mmol/L)**		3.26 ± 1.24	3.29 ± 1.23	2.86 ± 1.31	<0.001*	3.23 ± 1.28	3.25 ± 1.27	2.97 ± 1.34	<0.001*
**Triglyceride (mmol/L)**		1.71 ± 0.96	1.72 ± 0.96	1.54 ± 0.83	<0.001*	1.77 ± 1.12	1.78 ± 1.13	1.61 ± 1.00	<0.001*
**Fasting Blood Glucose (mmol/L) (fbg)**		8.28 ± 3.96	8.12 ± 3.74	11.14 ± 6.24	<0.001*	8.11 ± 3.95	7.98 ± 3.78	9.95 ± 5.42	<0.001*
**Electrocardiography**									
**Abnormalities type**									
**ST-segment Elevation ≥1mm in ≥ 2 Contiguous Limb Leads**					0.237				<0.001*
	1: Selected	3840 (29.1%)	3629 (29.0%)	211 (31.1%)		15992 (23.3%)	14474 (22.7%)	1518 (31.6%)	
	2: Not Selected	9350 (70.9%)	8883 (71.0%)	467 (68.9%)		52536 (76.7%)	49245 (77.3%)	3291 (68.4%)	
**ST-segment Elevation ≥ 2mm in ≥ 2 Contiguous Frontal Leads**					<0.001*				<0.001*
	1: Selected	4602 (34.9%)	4322 (34.5%)	280 (41.3%)		19303 (28.2%)	17372 (27.3%)	1931 (40.2%)	
	2: Not Selected	8588 (65.1%)	8190 (65.5%)	398 (58.7%)		49225 (71.8%)	46347 (72.7%)	2878 (59.8%)	
**ST-segment Depression ≥ 0.5mm in ≥ 2** **Contiguous Leads**					<0.001*				<0.001*
	1: Selected	2831 (21.5%)	2622 (21.0%)	209 (30.8%)		14912 (21.8%)	13583 (21.3%)	1329 (27.6%)	
	2: Not Selected	10359 (78.5%)	9890 (79.0%)	469 (69.2%)		53616 (78.2%)	50136 (78.7%)	3480 (72.4%)	
**T-wave inversion ≥1mm**					<0.001*				<0.001*
	1: Selected	2737 (20.8%)	2638 (21.1%)	99 (14.6%)		15730 (23.0%)	15129 (23.7%)	601 (12.5%)	
	2: Not Selected	10453 (79.2%)	9874 (78.9%)	579 (85.4%)		52798 (77.0%)	48590 (76.3%)	4208 (87.5%)	
**Bundle Branch Block (BBB)**					<0.001*				<0.001*
	1: Selected	476 (3.6%)	428 (3.4%)	48 (7.1%)		3048 (4.4%)	2664 (4.2%)	384 (8.0%)	
	2: Not Selected	12714 (96.4%)	12084 (96.6%)	630 (92.9%)		65480 (95.6%)	61055 (95.8%)	4425 (92.0%)	
**Abnormalities location**									
**Inferior Leads: II, III, aVF**					0.633				<0.001*
	1: Selected	4919 (37.3%)	4672 (37.3%)	247 (36.4%)		22240 (32.5%)	20446 (32.1%)	1794 (37.3%)	
	2: Not Selected	8271 (62.7%)	7840 (62.7%)	431 (63.6%)		46288 (67.5%)	43273 (67.9%)	3015 (62.7%)	
**Anterior Leads: V1 to V4**					<0.001*				<0.001*
	1: Selected	6074 (46.1%)	5692 (45.5%)	382 (56.3%)		27665 (40.4%)	25201 (39.6%)	2464 (51.2%)	
	2: Not Selected	7116 (53.9%)	6820 (54.5%)	296 (43.7%)		40863 (59.6%)	38518 (60.4%)	2345 (48.8%)	
**Lateral Leads: 1, aVL, V5 to V6 (ecgabnormlocationll)**					0.009				<0.001*
	1: Selected	4000 (30.3%)	3764 (30.1%)	236 (34.8%)		19703 (28.8%)	18080 (28.4%)	1623 (33.7%)	
	2: Not Selected	9190 (69.7%)	8748 (69.9%)	442 (65.2%)		48825 (71.2%)	45639 (71.6%)	3186 (66.3%)	
**True Posterior: V1, V2**					0.522				<0.001*
	1: Selected	707 (5.4%)	667 (5.3%)	40 (5.9%)		3087 (4.5%)	2797 (4.4%)	290 (6.0%)	
	2: Not Selected	12483 (94.6%)	11845 (94.7%)	638 (94.1%)		65441 (95.5%)	60922 (95.6%)	4519 (94.0%)	
**Right Ventricle: ST** **Elevation in Lead V4R**					0.576				<0.001*
	1: Selected	643 (4.9%)	613 (4.9%)	30 (4.4%)		2217 (3.2%)	1995 (3.1%)	222 (4.6%)	
	2: Not Selected	12547 (95.1%)	11899 (95.1%)	648 (95.6%)		66311 (96.8%)	61724 (96.9%)	4587 (95.4%)	
**Invasive Therapeutic Procedures**									
**Cardiac Catherization**					<0.001*				<0.001*
	1: Yes	5940 (45.0%)	5722 (45.7%)	218 (32.2%)		22611 (33.0%)	21391 (33.6%)	1220 (25.4%)	
	2: No	7250 (55.0%)	6790 (54.3%)	460 (67.8%)		45917 (67.0%)	42328 (66.4%)	3589 (74.6%)	
**Percutaneous Coronary Intervention (PCI)**					<0.001*				<0.001*
	1: Yes	4413 (33.5%)	4250 (34.0%)	163 (24.0%)		16322 (23.8%)	15387 (24.1%)	935 (19.4%)	
	2: No	8777 (66.5%)	8262 (66.0%)	515 (76.0%)		52206 (76.2%)	48332 (75.9%)	3874 (80.6%)	
**CABG**					0.588				0.598
	1: Yes	147 (1.1%)	138 (1.1%)	9 (1.3%)		702 (1.0%)	657 (1.0%)	45 (0.90%)	
	2: No	13043 (98.9%)	12374 (98.9%)	669 (98.7%)		67826 (99.0%)	63062 (99.0%)	4764 (99.1%)	
**Pharmacological Therapy (Medication)**									
**ASA**					<0.001*				<0.001*
	1: Yes	12901 (97.8%)	12253 (97.9%)	648 (95.6%)		65723 (95.9%)	61352 (96.3%)	4317 (90.9%)	
	2: No	289 (2.2%)	259 (2.1%)	30 (4.4%)		2805 (4.1%)	2367 (3.7%)	438 (9.1%)	
**GP Receptor Inhibitor (gpri)**					0.169				<0.001*
	1: Yes	273 (2.1%)	254 (2.0%)	19 (2.8%)		1417 (2.1%)	1262 (2.0%)	155 (3.2%)	
	2: No	12917 (97.9%)	12258 (98.0%)	659 (97.2%)		67099 (97.9%)	62445 (98.0%)	4654 (96.8%)	
**Unfractioned Heparin**					0.134				<0.001*
	1: Yes	1716 (13.0%)	1615 (12.9%)	101 (14.9%)		7578 (11.1%)	6875 (10.8%)	703 (14.6%)	
	2: No	11474 (87.0%)	10897 (87.1%)	577 (85.1%)		60950 (88.9%)	56844 (89.2%)	4106 (85.4%)	
**LMWH**					0.012				0.067
	1: Yes	3703 (28.1%)	3484 (27.8%)	219 (32.3%)		23507 (34.3%)	21785 (34.2%)	1722 (35.8%)	
	2: No	9487 (71.9%)	9028 (72.2%)	459 (67.7%)		45018 (65.7%)	41931 (65.8%)	3087 (64.2%)	
**Beta-blocker**					<0.001*				<0.001*
	1: Yes	8201 (62.2%)	8000 (63.9%)	210 (29.6%)		43651 (63.7%)	42220 (66.3%)	1431 (29.8%)	
	2: No	4989 (37.8%)	4512 (36.1%)	477 (70.4%)		24877 (36.3%)	21499 (33.7%)	3378 (70.2%)	
**ACE Inhibitors**					<0.001*				<0.001*
	1: Yes	6693 (50.7%)	6570 (52.5%)	123 (18.1%)		37072 (54.1%)	36059 (56.6%)	1013 (21.1%)	
	2: No	6497 (49.3%)	5942 (47.5%)	555 (81.9%)		31456 (45.9%)	27660 (43.4%)	3796 (78.9%)	
**Angiotensin II Receptor Blocker**					<0.001*				<0.001*
	1: Yes	680 (5.2%)	669 (5.3%)	11 (1.6%)		4748 (6.9%)	4580 (7.2%)	168 (3.5%)	
	2: No	12510 (94.8%)	11843 (94.7%)	667 (98.4%)		63780 (93.1%)	59139 (92.8%)	4641 (96.5%)	
**Statin**					<0.001*				<0.001*
	1: Yes	12513 (94.9%)	11913 (95.2%)	600 (88.5%)		63321 (92.4%)	59551 (93.4%)	3810 (79.2%)	
	2: No	677 (5.1%)	599 (4.8%)	78 (11.5%)		5207 (7.6%)	4208 (6.6%)	999 (20.8%)	
**Other Lipid Lowering Agent**					0.002				<0.001*
	1: Yes	313 (2.4%)	309 (2.5%)	4 (0.6%)		2535 (3.7%)	2438 (3.8%)	97 (2.0%)	
	2: No	12877 (97.6%)	12203 (97.5%)	674 (99.4%)		65993 (96.3%)	61281 (96.2%)	4712 (98.0%)	
**Diuretics**					<0.001*				<0.001*
	1: Yes	3426 (26.0%)	3107 (24.8%)	319 (47.1%)		19318 (28.2%)	17301 (27.2%)	2017 (41.9%)	
	2: No	9764 (74.0%)	9405 (75.2%)	359 (52.9%)		49210 (71.8%)	46418 (72.8%)	2792 (58.1%)	
**Calcium antagonist**					<0.001*				<0.001*
	1: Yes	1710 (13.0%)	1655 (13.2%)	55 (8.1%)		9673 (14.1%)	9321 (14.6%)	352 (7.3%)	
	2: No	11480 (87.0%)	10857 (86.8%)	623 (91.9%)		58855 (85.9%)	54398 (85.4%)	4457 (92.7%)	
**Oral hypoglycemic agent**					<0.001*				<0.001*
	1: Yes	3185 (24.1%)	3129 (25.0%)	56 (8.3%)		17057 (24.9%)	16568 (26.0%)	489 (10.2%)	
	2: No	10005 (75.9%)	9383 (75.0%)	622 (91.7%)		51471 (75.1%)	47151 (74.0%)	4320 (89.8%)	
**Insulin**					<0.001*				<0.001*
	1: Yes	3285 (24.9%)	3029 (24.2%)	256 (37.8%)		16925 (24.7%)	15277 (24.0%)	1648 (34.3%)	
	2: No	9905 (75.1%)	9483 (75.8%)	422 (62.2%)		51603 (75.3%)	48442 (76.0%)	3161 (65.7%)	
**Anti-arrhythmic Agent**					<0.001*				<0.001*
	1: Yes	541 (5.1%)	467 (3.7%)	74 (10.9%)		3967 (5.8%)	3346 (5.3%)	621 (12.9%)	
	2: No	12649 (95.9%)	12045 (86.3%)	604 (89.1%)		64561 (94.2%)	60373 (94.7%)	4188 (87.1%)	

Abbreviations: CAD = coronary artery disease, HDL = high-density lipoprotein, LDL = low-density lipoprotein, ECG = electrocardiogram, PCI = percutaneous coronary intervention, CABG = coronary artery bypass graft, ASA = acetylsalicylic acid (aspirin), GP = glycoprotein, LMWH = low molecular-weight heparin, ACE = Angiotensin-converting enzyme.

Note: The asterisk (*) with p-value <0.001 indicated that the variable difference between the alive and dead group is statistically significant.

#### Data splitting

**Fig 1:**
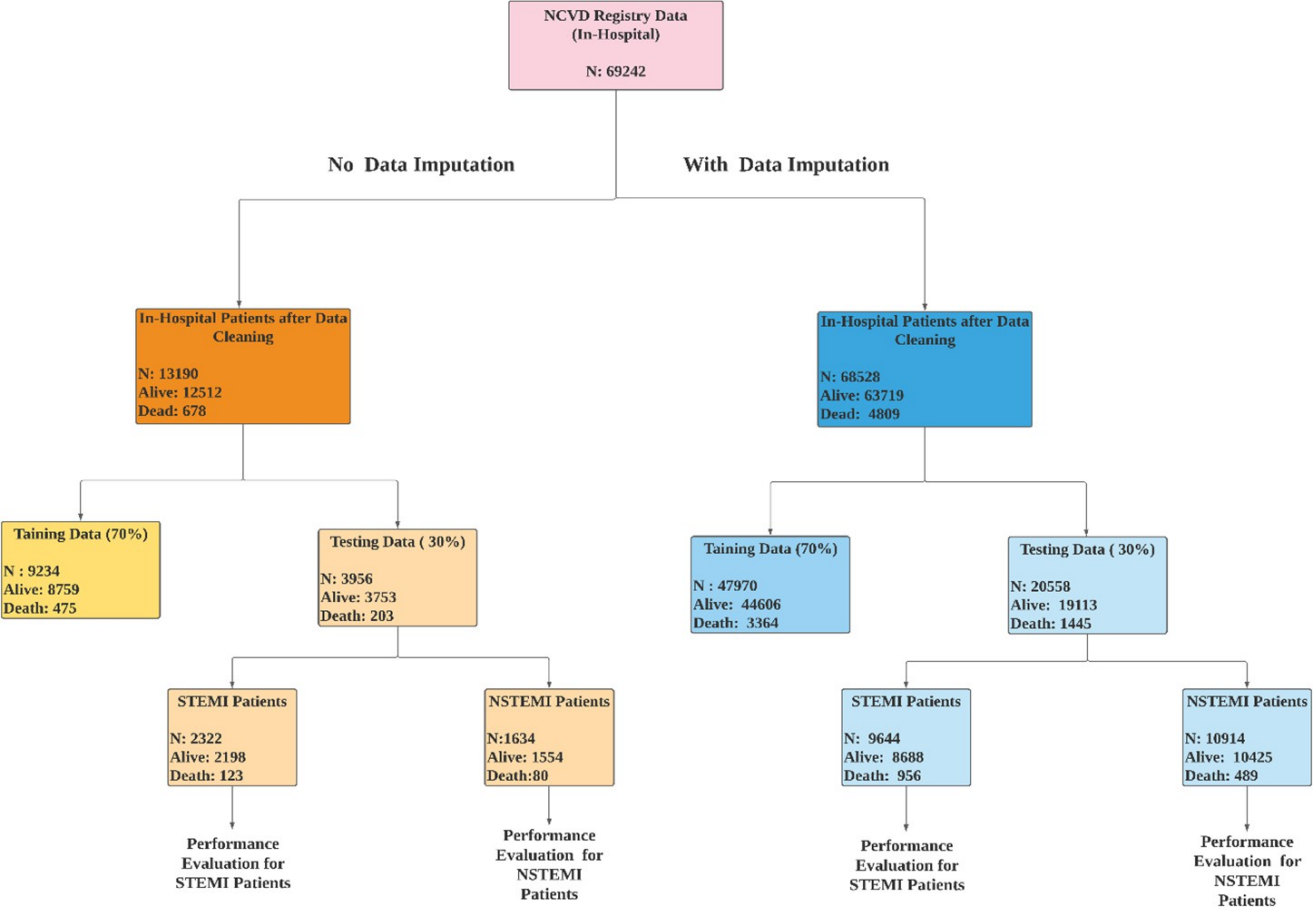
Study data details.

We used stratified random sampling of data [41]. Data were split for model development (70%) and validation (30%) for complete and missing cases which are shown in Fig 1 below. We accessed the performance of the developed model and TIMI using a validation set that accounts for 30% of data that was not used for model development. Fig 1 below shows the details of the study data.

#### Data normalization

Data normalization is a pre-processing step where data is scaled or altered to contribute equally to each feature. This reduces the bias of features that contribute more numerically to pattern class discrimination [42].

We employed standardization or z-score normalization, where values are centred around the mean with a unit standard deviation, resulting in a mean of zero with a unit standard deviation.

Using z-score normalization, continuous variables (age, heart rate, Systolic and Diastolic Blood Pressure, Peak CK, Total Cholesterol, HDL, LDL, Triglyceride, Fasting Blood Glucose were normalized.

#### Algorithm development and calibration

We used DL and ML classification methods, random forest (RF), support vector machine (SVM), and logistic regression (LR). They are the classifiers that outperform traditional approaches in mortality studies [20, 43]. K-fold cross validation was used to during the algorithm training the value of k was set to k = 5. Each algorithm was trained with all 54 variables and features obtained by sequential backward elimination features selection (SBE).

The DL and ML algorithms’ parameters were tuned for better prediction as referred to Table 1. Tuned hyperparameters are known to outperform the default settings in ML and DL models [44].

The area under the curve (AUC) was used to assess predictive performance [45]. Model calibration performance indicators were accuracy, sensitivity, specificity, positive predictive value (PPV), and negative predictive value (NPV) [46]. In addition, we used McNemar’s test, a non-parametric approach for testing row and column marginal frequencies [47].

The McNemar test can also be used to compare two groups on a dichotomous dependent variable. In contrast to independent data, McNemar’s test uses dependent (paired or correlated) data [48]. In addition, the paired resampled t-test was performed [41, 49]. Table 2 shows the hyperparameters used in ML Models Development as for Table 3 displays the hyperparameters used for all the DL models with all and selected features.

**Table 2:** Hyperparameters used in ML models development.

Models	LR	RF	SVM
**Method**	glm	rf	svmLinear
**Parameters Used**	**family** = “binomial”	**ntree** = 1000 **mtry** = 7	**Kernal Functions:** Linear **Best Cost Function (C):** 1

**Table 3:** Hyperparameters used for all the DL models with all and selected features.

Hyperparameters	Tuning Value Used			
**Models**	DL (all var)	DL (LR selected var)	DL (RF selected var)	DL (SVM selected var)
**Numbers of nodes**	246 (54,128,64,2)	68 (18,32,16,2)	70 (20,32,16,2)	72 (14,32,16,8,2)
**Numbers of hidden layers**	2	3	2	3
**Dropout rate**	0.2	0.2	0.2	0.2
**Learning rate**	0.001	0.001	0.001	0.01
**Optimizer**	Adagrad	Adagrad	Adagrad	Adagrad
**Batch size**	32	64	32	16
**Epoch**	200	200	200	200
**Activation Function**	relu for hidden layers sigmoid for output layer			

#### Feature selection

By removing duplicate, irrelevant, or noisy features from the original set of features, feature selection reduces dimensionality and improves learning performance [50].

Using classifier specific variable evaluators, we employed feature selection to rank variables. The relevance of variables related to outcome (in-hospital survival) was ranked using RF, SVM, and LR.

Then, sequential backward elimination (SBE) [51] was performed to reduce the number of features on the ML variables ranked in ascending order of relevance. Every time a variable is eliminated, the model is retrained and tested. The feature selection technique identifies the variable that reduces the AUC of the prediction model by a significant amount upon elimination. Next, we rank the selected variables again and resume the elimination procedure until we achieve a model with the least number of variables and the highest AUC. The DL algorithm was then trained using the final set of ML feature variables.

**Fig 2:**
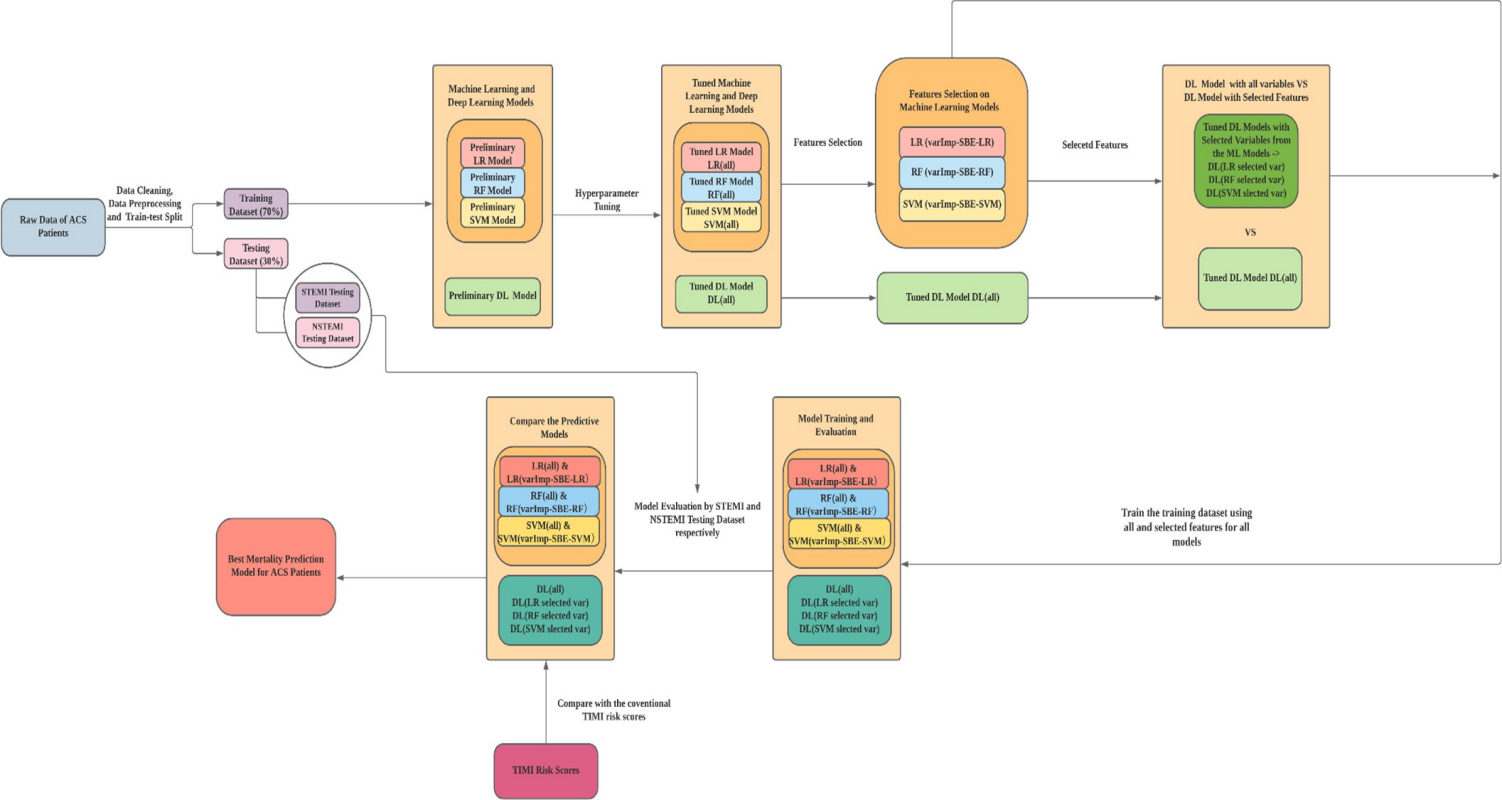
Flowchart of the predictive models’ development.

Shapley Additive Explanations (SHAP) were used to interpret our model because Shapley values are used to measure the contribution of input features to the output of a machine learning model at the instance level. These SHAP values encode the importance that a model assigns to a feature, so we can use them to order the features according to their importance [52]. A SHAP force plot was also used to show how features influenced the model’s prediction for a specific observation. This explains how the model came to make the prediction it did for a specific observation [53]. **Fig 2 illustrates the model development process in this study**.

#### Comparison with TIMI score

Performance was compared using NCVD registry calculated STEMI and NSTEMI TIMI scores. The AUC of the TIMI score was compared to the developed DL and ML-based models using the 30% validation set. Based on clinical and research cut off points, we created a graph to compare the best model and TIMI score [54]. A high risk of death was defined as a probability risk of death greater than 8%, as defined by Correia et al. [54].

The best model and TIMI risk scores were compared using net reclassification improvement (NRI). The NRI employs reclassification tables to analyse whether reclassifying patients using a different technique to mortality assessment adds value. The NRI allowed us to quantify how well the various mortality risk assessment methods drove correct category change. An NRI is the percentage improvement in net categorization employing a new approach. The NRI was calculated by comparing the TIMI risk score for STEMI and NSTEMI to the best model for STEMI and NSTEMI [55].

#### Additional statistics

The findings are provided as mean + SD, and categorical variables as frequency and percentage. We used correlation analysis to find a substantial association between variables. We used a Chi-Square test to identify significant variables and a two-sided independent student t-test (p = 0.05) to compare them. The t-test was used to compare the performance of all develop models [56, 57]. Statistical significance was defined as 0.05 or less.

#### Software

R package (Version 3.5.2) was used in DL and ML algorithm development. Statistical analysis was conducted using Statistical Package for Social Sciences (SPSS) program version 16.0 [58].

## Results

### Patient characteristics

A total of 68,528 ACS patients were identified. After removing patients with incomplete data, 68,528 patients were enrolled. Table 2 illustrates patients’ characteristics used in this study on the complete dataset and imputed dataset. The mean age was 58.42 (SD = 12.04). The majority of patients (~79.7%) were males. The overall mortality reported for in-hospital was 5.41%. STEMI patients (58.70%) and NSTEMI patients (41.30%) excluding the unstable angina patients (UA), made the complete case population dataset and for imputed dataset STEMI patients comprised of 46.91% and NSTEMI, 53.09%. There were significant differences between survivors to non-survivors for in-hospital, in terms of age, gender, smoking status, history of diabetes, hypertension, family history of premature cardiovascular disease, heart failure, renal disease, heart rate, history of cerebrovascular disease, heart rate, systolic blood pressure, diastolic blood pressure, Killip class, total cholesterol, LDL, triglyceride, fasting blood glucose, ECG abnormalities, cardiac catheterization, PCI, anterior leads, ASA, beta-blocker, ACE inhibitor, Angiotensin II Receptor Blocker, statin, diuretics, insulin, calcium antagonist, oral hypoglycemia and anti-arrhythmic agent use (p < 0.0001 for all). Both statistical analyses on the complete and imputed dataset are noted be almost similar.

**Fig 3:**
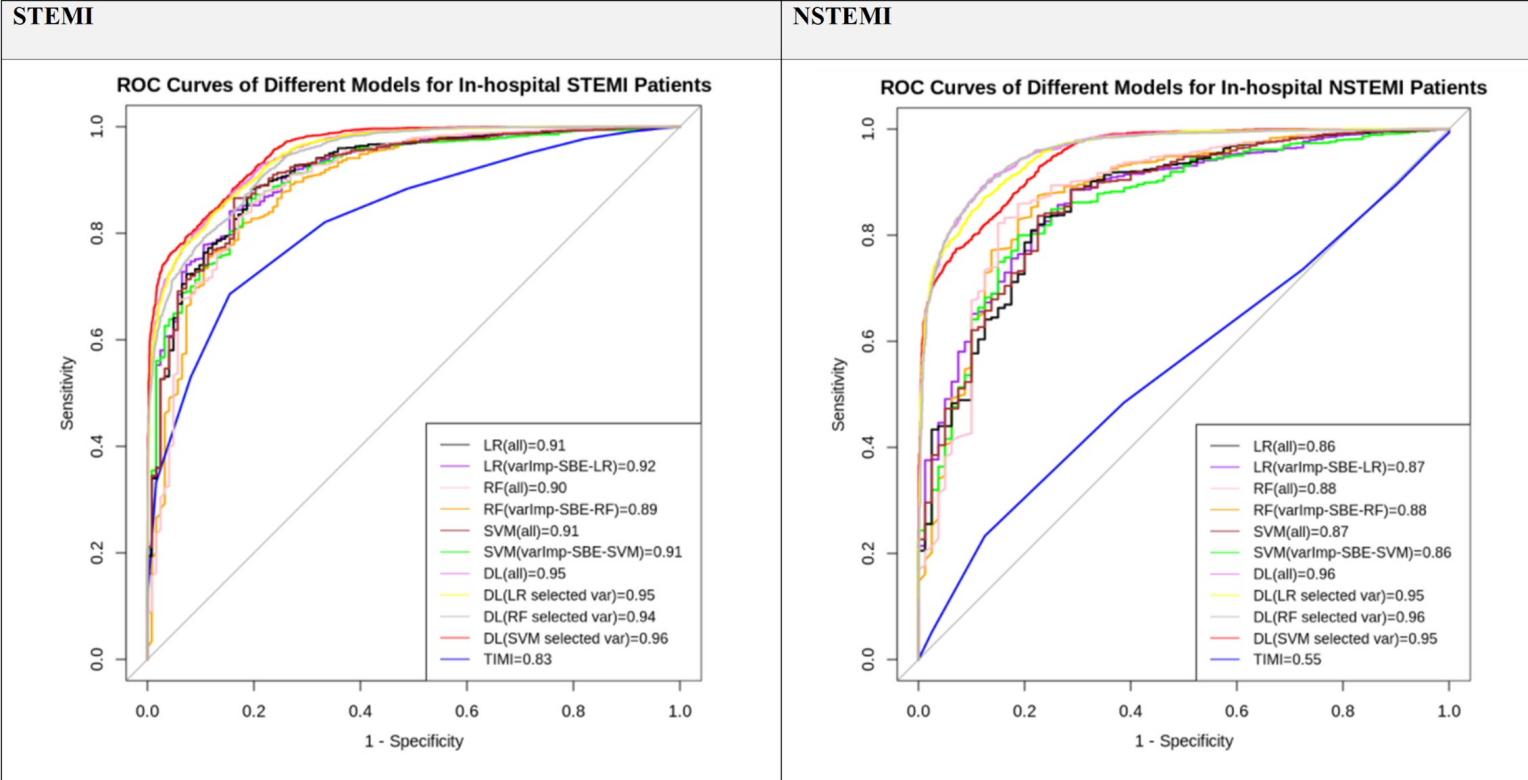
ROC curves for in-hospital mortality prediction for STEMI and NSTEMI patients.

Maximal predictive performances on the 30% testing dataset were observed for the models constructed using complete sets (54 variables) and a reduced set of variables compared to the TIMI risk score as shown in Table 4. All the DL and ML models outperformed TIMI risk scores in both STEMI and NSTEMI prediction which depicts in Fig 3. The best-selected model was DL (SVM selected var) (p<0.0001). Detailed performance evaluation of the DL and ML model against the TIMI risk score is presented in Table 5.

**Table 4:** The AUC of TIMI risk score, DL and ML models with and without feature selection based on a 30% validation dataset.

Models	The area under the ROC Curve (95% CI)	
	STEMI	NSTEMI
**LR (all)**	0.91 (0.89,0.94)	0.86 (0.82,0.92)
**LR (varImp—SBE-LR)**	0.92 (0.89,0.94)	0.87 (0.83,0.91)
**RF (all)**	0.90 (0.87,0.93)	0.88 (0.83,0.92)
**RF (varImp-SBE-RF)**	0.89 (0.89,0.93)	0.88 (0.88,0.92)
**SVM (all)**	0.91 (0.89,0.94)	0.87 (0.82,0.91)
**SVM (varImp-SBE-SVM)**	0.91 (0.91,0.93)	0.86 (0.86,0.90)
**DL (all)**	0.95 (0.94,0.96)	0.96 (0.95,0.97)
**DL (LR selected var)**	0.95 (0.94,0.96)	0.95 (0.95,0.96)
**DL (RF selected var)**	0.94 (0.93,0.95)	0.96 (0.95,0.96)
**DL (SVM selected var)**	0.96 (0.95,0.96)	0.95 (0.94,0.95)
**TIMI**	0.83 (0.80,0.86)	0.55 (0.39,0.51)

Abbreviations:

LR (all) = LR with all variables

LR (varImp—SBE-LR) = LR variable importance with sequential backward elimination and LR classifier

RF (all) = RF with all variables

RF (varImp-SBE-RF) = RF variable importance with sequential backward elimination and RF classifier

SVM (all) = SVM with all variables

SVM (varImp-SBE-SVM) = SVM variable importance with sequential backward elimination and SVM classifier

DL (all) = DL with all variables

DL (LR selected var) = DL with LR with selected variables

DL (RF selected var) = DL with RF with selected variables

DL (SVM selected var) = DL with SVM selected variables

**Table 5:** Detailed performance metrics of DL and ML with and without feature selection for STEMI and NSTEMI patients.

Models	STEMI						NSTEMI					
	Accuracy (95% CI)	Sensitivity	Specificity	PPV	NPV	Mcnemar’s test (p-value)	Accuracy (95% CI)	Sensitivity	Specificity	PPV	NPV	Mcnemar’s test (p-value)
**LR (all)**	0.83 (0.81,0.84)	0.83	0.83	0.21	0.99	<0.0001	0.86 (0.84,0.87)	0.71	0.86	0.21	0.98	<0.0001
**LR (varImp-SBE-LR)**	0.84 (0.83,0.86)	0.85	0.84	0.23	0.99	<0.0001	0.84 (0.82,0.86)	0.73	0.85	0.20	0.98	<0.0001
**RF (all)**	0.95 (0.94,0.96)	0.49	0.98	0.57	0.97	<0.1	0.96 (0.94,0.96)	0.26	0.99	0.58	0.96	<0.0001
**RF (varImp-SBE-RF)**	0.94 (0.93,0.95)	0.53	0.96	0.44	0.97	<0.1	0.95 (0.94,0.96)	0.32	0.98	0.45	0.97	<0.01
**SVM (all)**	0.83 (0.81,0.84)	0.84	0.83	0.21	0.99	<0.001	0.86 (0.84,0.87)	0.71	0.86	0.21	0.98	<0.001
**SVM (varImp-SBE-SVM)**	0.83 (0.81, 0.84)	0.82	0.83	0.21	0.99	<0.0001	0.83 (0.81,0.85)	0.75	0.83	0.19	0.95	<0.0001
**DL (all)**	0.85 (0.84,0.87)	0.79	0.86	0.24	0.99	<0.0001	0.89 (0.87,0.90)	0.66	0.90	0.25	0.98	<0.0001
**DL (LR selected var)**	0.86 (0.84,0.87)	0.78	0.86	0.24	0.99	<0.0001	0.87 (0.85,0.89)	0.63	0.88	0.22	0.98	<0.0001
**DL (RF selected var)**	0.84 (0.82,0.85)	0.80	0.84	0.22	0.99	<0.0001	0.88 (0.87,0.90)	0.68	0.89	0.25	0.98	<0.0001
**DL (SVM selected var)**	0.86 (0.84,0.87)	0.81	0.86	0.25	0.99	<0.0001	0.85 (0.83,0.86)	0.70	0.86	0.20	0.98	<0.0001

### Performances evaluation

DL and ML algorithms constructed using all and selected features outperformed TIMI risk scores for both STEMI and NSTEMI predictions on the 30% untouched validation dataset (p < 0.0001).

The DL (SVM selected var) algorithm demonstrated the highest predictive performance with the least number of predictors (14 predictors) for in-hospital prediction of STEMI patients (AUC = 0.96, 95% CI: 0.95–0.96). In NSTEMI in hospital prediction, DL (all) without feature selection (AUC = 0.96, 95% CI: 0.95–0.97) reported similar performance with DL (RF selected var) (AUC = 0.96, 95% CI: 0.95–0.96, p < 0.0001) and slightly higher AUC compared to DL (SVM selected var) (AUC = 0.95, 95% CI: 0.94–0.95). There was no significance difference between DL (SVM selected var) algorithm and DL (RF selected var) algorithm (p = 0.5).

However, the DL (SVM selected var) model consisted of the least number of predictors (14 predictors) compared to DL (all) without feature selection (54 predictors), DL (RF selected var) (20 predictors) and DL (LR selected var) (18 predictors).

### Results of data imputation

Secondary analysis was conducted on the best performing algorithm with the least number of predictors DL (SVM selected var). The DL (SVM selected var) algorithm was trained and tested on the imputed dataset using two different imputation methods, MissForest and pmm are shown in Table 6. The DL (SVM selected var) on complete cases performed slightly better than the imputed model for STEMI and NSTEMI patients (p<0.0001). Similar performance was reported on the imputed dataset using both methods (p<0.0001).

**Table 6:** Detailed performance metrics of best DL model on an imputed dataset for STEMI and NSTEMI patients.

	STEMI						NSTEMI					
	AUC (95% CI)	AUC (95% CI)	Sensitivity	Specificity	PPV	NPV	AUC (95% CI)	AUC (95% CI)	Sensitivity	Specificity	PPV	NPV
**DL (SVM selected var)**	0.96 (0.95,0.96)	0.86 (0.84,0.87)	0.81	0.86	0.25	0.99	0.95 (0.94,0.95)	0.85 (0.83,0.86)	0.70	0.86	0.20	0.98
**DL (SVM selected var) MICE**	0.93 (0.92, 0.93)	0.83 (0.83, 0,84)	0.70	0.85	0.34	0.96	0.95 (0.94,0.95)	0.87 (0.86, 0.87)	0.56	0.88	0.18	0.98
**DL (SVM selected var) MissForest**	0.93 (0.92,0.93)	0.82 (0.81, 0.83)	0.74	0.83	0.32	0.97	0.95 (0.94, 0.95)	0.85 (0.84,0.85)	0.63	0.85	0.17	0.98

### Feature selection

SBE feature selection methods were combined with ML algorithms to construct predictive models with optimal performance (refer to methods). Table 7 illustrates final predictors ranked in ascending order of importance. Common predictors observed for in-hospital, across all ML feature selection models in this study are age, heart rate, Killip Class, cardiac catheterization, oral hypoglycaemic agents and antiarrhythmic agent. The best model DL (SVM selected var) were constructed using 14 features selected from SVM (varImp-SBE-SVM) (Table 7). Common features between TIMI risk score for STEMI and NSTEMI and the features from the best model are age, heart rate, Killip Class, fasting blood sugar and angina.

**Table 7:** Selected variables for each ML model ranked in ascending order that resulted in optimum AUC for in-hospital, against TIMI risk score for STEMI and NSTEMI variables.

Variables Importance	LR (varImp- SBE-LR) (18 Variables)	RF (varImp- SBE-RF) (20 Variables)	SVM (varImp- SBE-SVM) (14 Variables)
**1**	***Killip class***	***Killip Class***	***Killip class***
**2**	***Age***	***Heart rate***	***Fasting Blood Glucose***
**3**	***Heart Rate***	***Age***	***Heart Rate***
**4**	Systolic blood pressure	***Fasting Blood Glucose***	***Age***
**5**	***Fasting Blood Glucose***	Creatine kinase	Low density Lipoprotein
**6**	Beta blocker	Systolic blood pressure	Oral hypoglycemic agent
**7**	Oral hypoglycemic agent	Total Cholesterol	***Cardiac catheterization***
**8**	***Cardiac catheterization***	Low density Lipoprotein	High Density Lipoprotein
**9**	ST-segment Depression ≥ 0.5mm in ≥ 2 Contiguous Leads	Beta blocker	***Antiarrhythmic agent***
**10**	Coronary artery bypass grafting	ACE inhibitors	Statin
**11**	ST-segment Elevation ≥1mm in ≥ 2 Contiguous Limb Leads	Oral hypoglycemic agent	Chronic angina past 2 weeks
**12**	***Antiarrhythmic agent***	***Cardiac catheterization***	Lipid lowering agent
**13**	Statin	History of hypertension	ST-segment Elevation ≥1mm in ≥ 2 Contiguous Limb Leads
**14**	Chronic angina past 2 weeks	Documented coronary artery disease	Coronary artery bypass grafting
**15**	High Density Lipoprotein	***Antiarrhythmic agent***	
**16**	Anterior Leads: V1 to V4	Heparin	
**17**	Triglyceride	History of heart failure	
**18**	Bundle branch block	Angiotensin II Receptor Blocker	
**19**		GP receptor inhibitors	
**20**		History of peripheral vascular disease	

*Variables in Blue: Predictors in common among all the 3 models after features selection.

**Fig 4:**
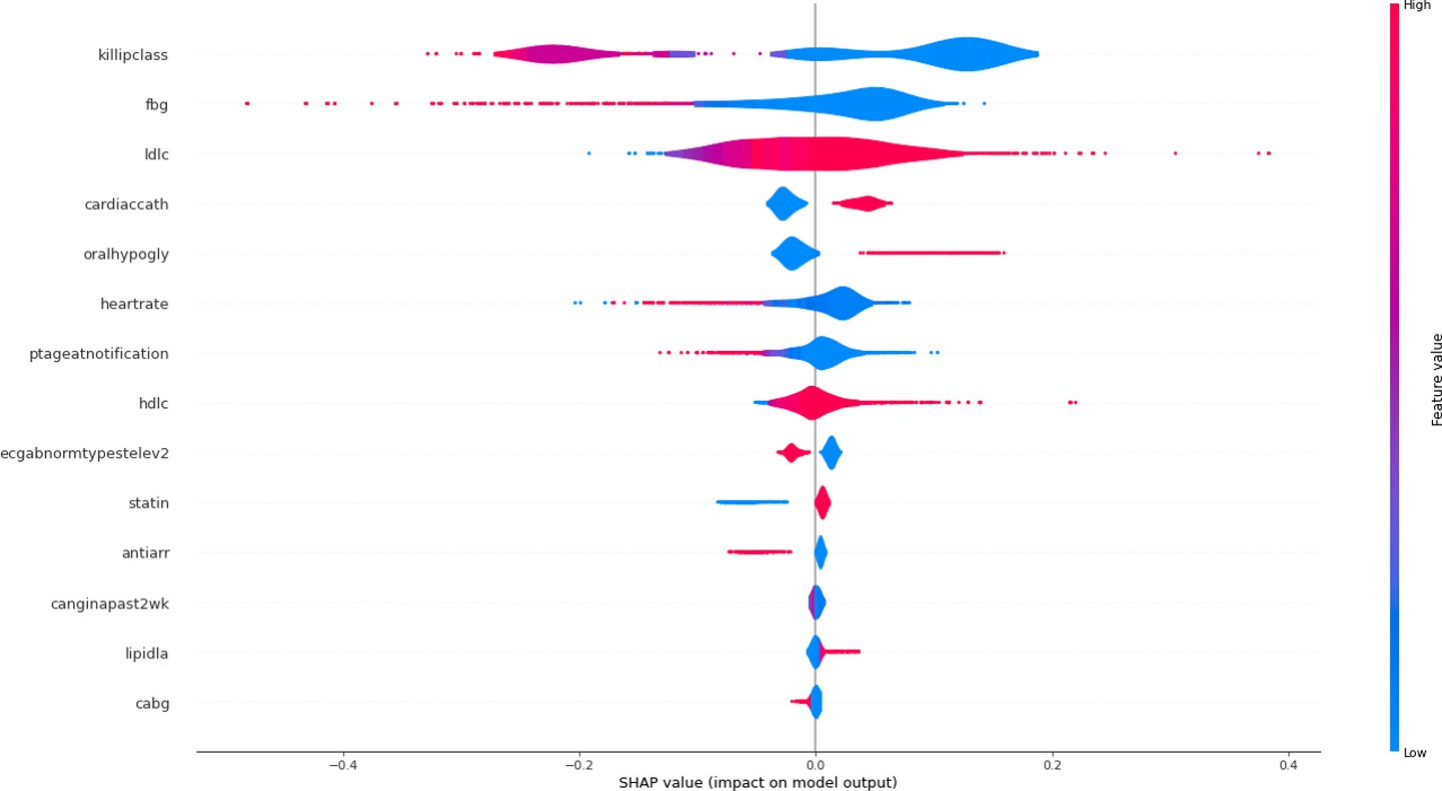
SHAP summary plot of SVM (varImp-SBE-SVM).

Fig 4 depicts the SHAP summary plot of the SVM (varImp-SBE-SVM) predictors that were combined with DL to achieve the highest performance accuracy.

The y-axis indicates the variable name in descending order of importance, with Killip class having the highest importance. On the x-axis is the SHAP value. The gradient colour indicates the variable’s initial value. In Booleans, it can contain only two colours, but in numbers, it can contain the entire colour spectrum. Each point corresponds to a row in the initial dataset.

Having a high Killip class, heart rate, age, and fasting blood glucose is associated with high and negative values on the outcome, as observed. Where high is determined by the colour and negative by the x-value.

In other words, as the Killip class, age, fasting blood glucose (FBG), and heart rate increase, so does the mortality rate. Meanwhile, cardiac catheterization and medications like oral hypoglycemic agents, as well as high HDL levels, are linked to survival or a favorable outcome. In the acute setting, such as ACS, LDL-C appears to have a more neutral effect, with high values contributing to similar outcomes.

**Fig 5:**
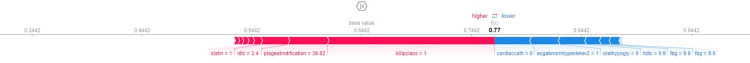
SHAP force plot.

The SHAP force plot in Fig 5 illustrate explanation of the DL for one single observation. The binary goal is either survival (survival = 1) or non-survival (non-survival = 0). The bold value **0.77** in the plot above represents the model’s score for this observation. Higher scores cause the model to predict 1, while lower scores cause it to predict 0. The features that were important in making the prediction for this observation are shown in red and blue, with red representing features that increased the model score and blue representing features that decreased it. Features that had a greater impact on the score are located closer to the red-blue dividing line, and the size of that impact is represented by the size of the bar.

As can be seen, the patient Killip class, age at onset of ACS, history of taking statins, and LDL Cholesterol values have a stronger association with a poorer outcome, i.e. death, which is similar to what is seen using traditional risk prediction methods. What’s interesting is that variables like whether the patient had an in-patient cardiac catheterization, an abnormal ECG on admission, a history of diabetes and taking oral hypoglycemics, as well as high HDL cholesterol and fasting blood sugar, all help improve the algorithm’s prediction of events, resulting in a better AUC with our algorithm.

The best model DL (SVM selected var) was converted into an in-hospital ACS online mortality calculator available at http://myheartstemiacs.uitm.edu.my/home.

### Comparison of best model DL (SVM selected var) to TIMI risk score when applied to the validation dataset

**Fig 6:**
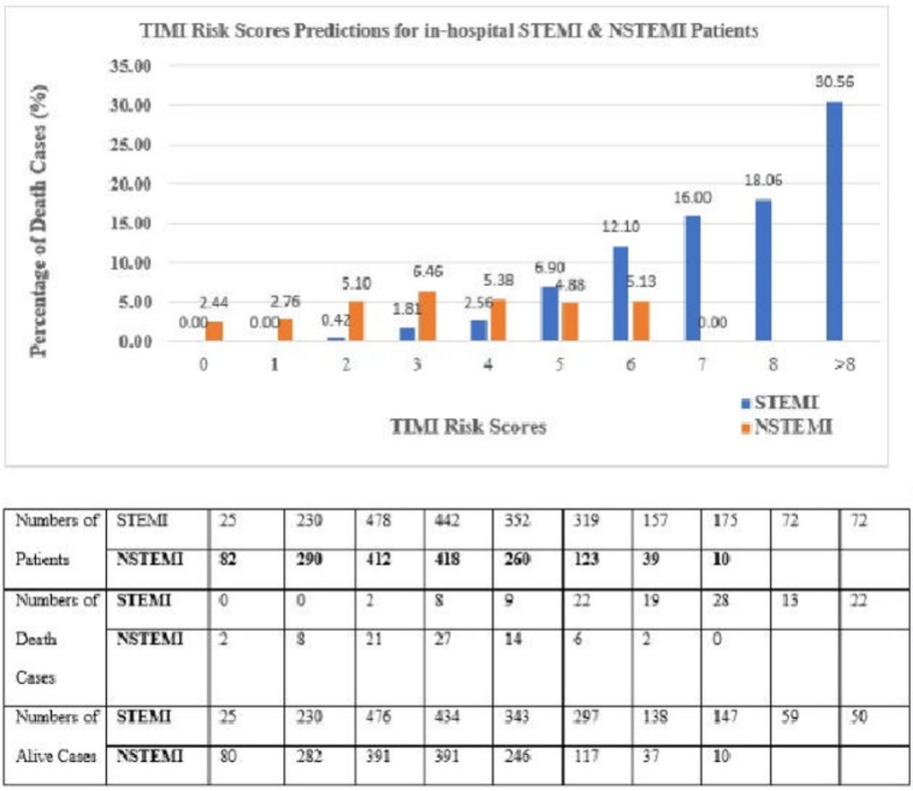
Breakdown of performance by the TIMI model for in-hospital mortality prediction for both STEMI and NSTEMI patients.

**Fig 7:**
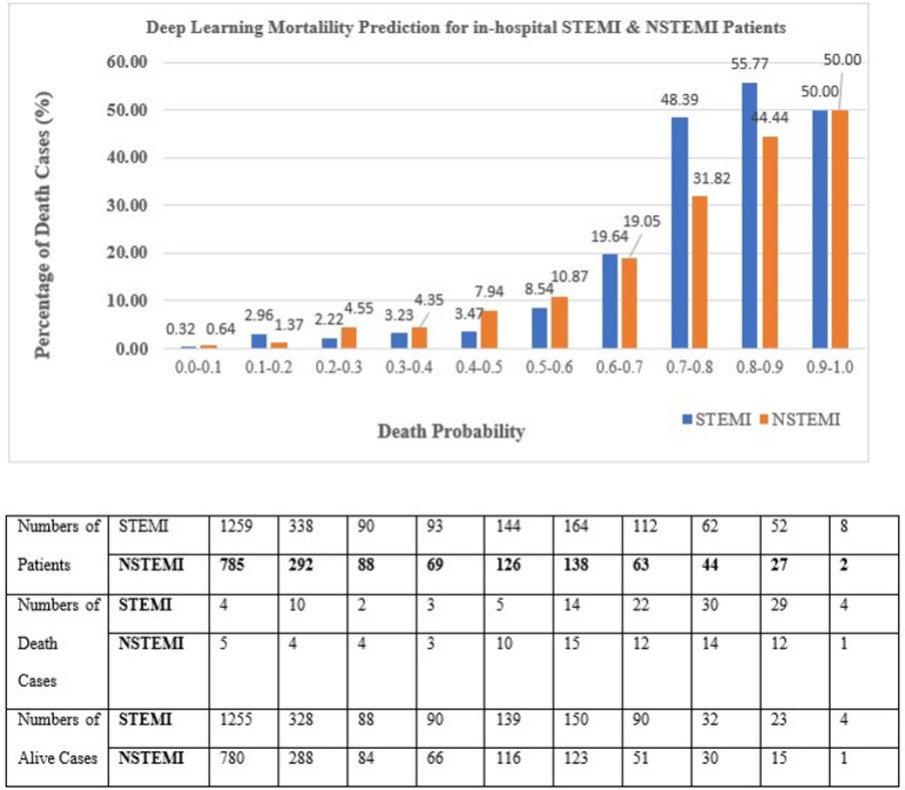
Breakdown of performance for DL (SVM selected var) in- hospital mortality prediction for both STEMI and NSTEMI patients.

Figs 6 and 7 illustrate the comparison of the best DL (SVM selected var) model mortality rate against the TIMI score for STEMI and NSTEMI. TIMI Risk Score for STEMI has a scale of 0–14 while TIMI Risk Score for NSTEMI has a scale of 0–7. We categorized DL score patients as low risk with the probability <50% and high-risk stratum as ≥50%. This is equivalent to TIMI low risk of score ≤5 and a high-risk score of > 5 for both STEMI and NSTEMI risk scores (5) (4).

The TIMI risk scores for STEMI correctly predicted 13.08% of the high-risk patient’s STEMI death, whereas the TIMI risk scores for NSTEMI only predicted 4.65% of the high-risk patient’s NSTEMI death (illustrates in Fig 4). In both STEMI and NSTEMI cases, the prediction using TIMI risk scores shows a poor prediction for the mortality rate of high-risk patients. The STEMI prediction model depicts an increasing trend, whereas the NSTEMI prediction model depicts a fluctuating trend.

Meanwhile, Fig 6 demonstrates the percentage of mortalities estimated at various probabilities using DL (SVM selected var) as the predictive model on the validation dataset.

The DL (SVM selected var) model correctly classified 24.87% of STEMI patients and 19.71% of NSTEMI patients as high risk (risk probabilities greater than 50%). When compared to the TIMI risk score, the DL (SVM selected var) classified a higher percentage of STEMI and NSTEMI high-risk patients.

### Net Reclassification Index (NRI)

NRI for the in-hospital model, the net reclassification of STEMI patients using the DL (SVM selected var) is shown in Table 8. DL produced a net reclassification improvement of **18.14%** with p<0.00001 over the original TIMI risk score. NRI for NSTEMI (Table 9) reported the net reclassification of patients improved using the DL (SVM selected var) produced a net reclassification improvement of 55.80% with p<0.00001 over the original TIMI risk score.

**Table 8:** NRI for STEMI patients.

STEMI						
**Individuals with Events (Died) (n = 123)**						
		Number of Individuals		Reclassification		Net Correctly Reclassified (%)
		Deep Learning		Increased Risk	Decreased Risk	13.82%
		Low Risk	High Risk	17	0	
	TIMI Score					
	Low Risk	**24**	**17**			
	High Risk	**0**	**82**			
**Individuals without Events (Alive) (n = 2199)**						
		Number of Individuals		Reclassification		Net Correctly Reclassified (%)
		Deep Learning		Increased Risk	Decreased Risk	4.32%
		Low Risk	High Risk	0	95	
	TIMI Score					
	Low Risk	**1805**	**0**			
	High Risk	**95**	**299**			
**Net Reclassification Index (NRI)**	**4.32 + 13.82 = 18.14**					
**Z, p-value**	**Z = $\frac{18.14}{\sqrt{\frac{17+0}{123^{2}}+\frac{0+95}{2199^{2}}}}$ = 536.48 536.48, p < 0.00001**					
**Conclusion**	It was statistically significant. The predictive power of the DL model was improved as compared to the TIMI Risk Scores Model in predicting mortality rate of NSTEMI patients, and the proportion of correct classification increased by **18.14%.**					

**Table 9:** NRI for NSTEMI patients.

NSTEMI						
**Individuals with Events (Died) (n = 80)**						
		Number of Individuals		Reclassification		Net Correctly Reclassified (%)
		Deep Learning		Increased Risk	Decreased Risk	65.00%
		Low Risk	High Risk	52	0	
	TIMI Score					
	Low Risk	**26**	**52**			
	High Risk	**0**	**2**			
**Individuals without Events (Alive) (n = 1554)**						
		Number of Individuals		Reclassification		Net Correctly Reclassified (%)
		Deep Learning		Increased Risk	Decreased Risk	-9.20%
		Low Risk	High Risk	143	0	
	TIMI Score					
	Low Risk	**1334**	**143**			
	High Risk	**0**	**47**			
**Net Reclassification Index (NRI)**	**65.00 + (-9.20) = 55.80**					
**Z, p-value**	**Z = $\frac{55.80}{\sqrt{\frac{52+0}{80^{2}}+\frac{143+0}{1554^{2}}}}$ = 616.80 616.80, p < 0.00001**					
**Conclusion**	It was statistically significant. The predictive power of the DL model was improved as compared to the TIMI Risk Scores Model in predicting mortality rate of NSTEMI patients, and the proportion of correct classification increased by **55.80%.**					

## Discussions

Our study is the first to demonstrate improved in-hospital mortality prediction in a multi-ethnic Asian patient with ACS that used a combination of DL and ML feature selection methods. On validation datasets, we demonstrated high performance for DL models using a combination of feature selection and ML classifier algorithms. Overall, the DL model, both with and without feature selection, outperformed the ML and TIMI risk scores for STEMI and NSTEMI in-hospital mortality. The best model identified in this study is the combination DL (SVM selected var) using 14 predictors with AUC of (STEMI = 0.96, NSTEMI = 0.95) for in-hospital ACS mortality prediction that resulted in better performance compared to other combinations of DL with ML and TIMI scoring as well. DL has proven to be better to ML in mortality studies using datasets of smaller or equal size to our study, achieving a higher AUC [7, 25, 59]. Conventional risk scoring such as TIMI uses logistic regression with few predictive parameters. The logistic regression model has two flaws: fixed assumptions on data behavior and the requirement to pre-select predictors during model development [7, 11, 60, 61].

On the study dataset, combining features selected by the SVM algorithm with a DL classifier produced high performance to other ML algorithms selected features. Similar publications have been published demonstrating that the SVM algorithm with features selection outperformed other ML algorithms [38] and when utilizing similar population datasets as demonstrated in our study [28, 62].

The TIMI score’s simplicity is recognized in current guidelines and is frequently used in Asian hospitals for risk assessment of patients with ACS. The TIMI risk score, originally established to predict 30-day mortality, is used in Asian hospitals to predict in-hospital, 30-day, and 1-year mortality post ACS [39, 63–65]. Correia et al. reported that the TIMI score is better than GRACE score calibration because it has more variables associated with mortality, a balanced distribution of low, intermediate, and high-risk patients, and more accurate estimation [56].

However, the TIMI score has several notable limitations. First, TIMI was developed using data from fibrinolytic-eligible patients with STEMI where reperfusion therapy and drug-eluting stents were not regular treatments [66]. Statins and antiplatelet medicines like prasugrel and ticagrelor are now part of our everyday routine. Because TIMI risk scores only reflect the key prognostic indicators, valuable information may be missed [7]. Exclusion of the high-risk patients is also another limitation of the risk score [33]. The TIMI risk score lacks risk factors relevant to older adults and fails to account for the overall complexity of the older adult with ACS. The Asian cohort was found to be carrying an overall higher disease burden and risk compared to the TIMI cohort.

The lack of weighting for the risk factors, while improving usability, decreased TIMI risk score discriminatory performance [6, 67]. In addition, there are different scoring systems for STEMI and NSTEMI.

To determine predictive mortality for ACS patients is important to strategize treatment plan and to improve outcomes. The database used for this study is unique in that it includes the three major ethnicities in Asia: Chinese, Indian, and Malay. Previous research relied on a homogeneous population database, raising concerns about its applicability to the Asian continent. The risk stratification model used in this study was developed using relatively recent data on Asian ACS patients, and it can better predict ACS patient mortality in modern practice. Different algorithms for the scoring method are eliminated for the status of the ST segment; moreover, the developed algorithm in our study can predict the mortality of ACS regardless of ST elevation.

Despite the fact that the TIMI risk score is widely used in the Asian population, it was developed using data from a Western Caucasian cohort with limited data from an Asian population. A previous validation study in the Asian population reported a modest accuracy for risk prediction for TIMI risk score in STEMI with an AUC of 0.78 [68]. Other conventional risk scores also performed modestly when validated in Korean registry study for STEMI and NSTEMI patients using AUC as a performance metric GRACE (0.851 0.810), ACTION (0.852, 0.806) and TIMI score (0.781, 0.593). In this study TIMI score validation for STEMI and NSTEMI resulted in (0.83, 0.55), implying similar performance for predicting the mortality of ACS patients in the Asian region.

As a result, risk scoring tools should be tailored to a specific population to more accurately reflect differences. In this study, we found that TIMI underestimated mortality risk in both lower and higher risk groups. This may cause treatment to be delayed, increasing avoidable deaths.

The net reclassification improvement of STEMI patients using the DL (SVM selected var) produced a net reclassification improvement of **18.14%,** and NSTEMI produced a net reclassification improvement of **55.80%** with respect to the original TIMI risk score. Despite its low NRI value for STEMI patients, we can see that significant improvement is added to the NSTEMI population, a cohort that accounts for half or more of all ACS cases worldwide.

We have included feature selection algorithms in this study to identify factors associated with mortality in Asian ACS patients. DL (SVM selected var) predictive performance requires only 14 predictors for in-hospital mortality prediction than models developed using a conventional statistical approach. TIMI requires two distinct scores; TIMI for STEMI 8 risk factors include age, systolic blood pressure, heart rate, Killip class, anterior or left bundle infarction, prior history of angina, diabetes, or hypertension, and weight. Meanwhile, the TIMI Risk Score for patients with UA or NSTEMI is composed of seven equally weighted, binary variables [69]. Age, aspirin use during the previous seven days, coronary artery disease (CAD) risk factors, known CAD, recent anginal episodes; ST-segment alterations of at least 0.5mm on the ECG at the time of initial presentation, and elevation of serum cardiac markers [67].

DL (SVM selected var) model with best performance in this study used 14 variables that include age, heart rate, Killip Class, fasting blood sugar, and angina low-density lipoprotein, high-density lipoprotein, statin, lipid-lowering drug, chronic angina episode, ST-segment Elevation ≥1mm in ≥ 2 contiguous limb leads, and coronary artery bypass grafting. The age, heart rate, Killip Class, fasting blood sugar, and angina are all shared characteristics between the TIMI risk score for STEMI and NSTEMI and the best model DL (SVM selected var). Previous studies using ML and DL algorithms identified significant predictors of mortality being age, Killip class, fasting blood glucose, heart rate, low-density lipoprotein, high-density lipoprotein, statin, ST-segment Elevation ≥1mm in ≥ 2 contiguous limb leads, and coronary artery bypass grafting were used as input predictors for STEMI and ACS patients [7,38, 60, 70].

We also performed univariate analysis to demonstrate the association between the variables chosen by the ML algorithm and the outcomes (Table 1). All ML feature selection models in this study selected age, heart rate, Killip class, fasting blood glucose, oral hypoglycemic drug, antiarrhythmic agent, and cardiac catheterization.

Using SHAP analysis to visualize the importance of selected variables allows us to understand and make logical inferences about how these variables were chosen as well as their impact on outcomes for the best model. According to the SHAP analysis (Fig 3), variables with higher Killip class, age, fasting blood glucose, and heart rate are all associated with a poorer outcome or non-survival. This is reported in the literature using conventional statistical methods [71, 72]. Our algorithm was able to add other variables that have significant values to outcomes, such as the presence of in-patient cardiac catheterization, having an abnormal ECG on admission, and the use of oral hypoglycemics.

This finding is novel because conventional approaches have identified only advanced age and a higher Killip class as significant factors in ACS patient mortality [73]. Incorporating invasive or non-invasive management into the DL (SVM selected var) model for in-hospital mortality prediction produced noteworthy findings. Invasive intervention, such as cardiac catheterization, was associated with improved outcomes in-hospital in STEMI patients [68, 74, 75]

TIMI and GRACE scores were generated using data collected before early reperfusion treatment and drug-eluting stents were routine. In our study, non-invasive treatment predictors associated with ACS mortality were selected included pharmacological therapy such as statin, oral hypoglycemic agents, antiarrhythmic medications, and lipid-lowering drugs. LDL-C is an independent CV risk factor, and more Asian individuals with a very high risk of recurrent cardiovascular events had LDL-C levels above the suggested range [17, 19]. The TG to HDL-C ratio is also a powerful independent predictor of all-cause death and a cardiovascular risk factor [76]. Thus, intensive lipid-lowering medication is required in ACS patients [18]. Lipid-lowering therapy was common but not fully utilized throughout Asia [17]. Statins are the foundation of lipid-lowering treatment in patients with ACS [19, 24].

Data imputation was performed to ensure the validity of the findings. We used two different types of imputation, MissForest and multivariable imputation using chained equations and predictive mean matching method for data imputation. MissForest is a machine learning-based method in this study [14]. The multivariable imputation using chained equations and predictive mean matching method [15] used in this study was selected as recommended in a similar study conducted on the Swedish heart registry dataset that resulted in high model performance [38].

Both data imputation methods produced a comparable predictive performance with the model built using complete cases. We initially did not include patients with more than 50% missing data because it would necessitate data imputation, which could affect our results. Because it is still a large dataset, we do not believe it is a limitation for the population. Because the dataset contained complete datasets for all follow-up time points, both the DL and TIMI calculators could be generated. Identifying factors associated with in-hospital ACS mortality prediction using complete cases, on the other hand, would result in more reliable findings. We returned to using an incomplete dataset and imputed data, and the results were similar.

Cross-validation and hyperparameter tuning improved model performance and reduced over-fitting risk [16, 77]. A pair-wise corrected resampled t-test was employed to compare model predictions [56, 57].

To ensure the current study’s reliability, all models were validated using untouched validation data that was not used for model construction. Additionally, we demonstrated the DL model utilizing complete sets of collected variables, without a variable selection method, and found that it performed similarly to models with feature selection. This demonstrates that feature selection does not result in the loss of critical prognostic information, as Kwon et al., 2019 assert [7].

Despite a high percentage of missing values in the original dataset, we were nevertheless able to apply and compare the TIMI and DL algorithms. This is most likely because we chose a fixed endpoint of death that is unaffected by missing numbers. Another argument is that the extracted variables were adequate to improve the model’s precision enough to consistently predict death.

By collecting continuous data via an electronic health record system, we were able to adapt DL and ML-based predictive algorithms to each patient’s risk categorization. The study’s findings also indicate that ML methods are required to rank and choose major risk factors linked with in-hospital ACS mortality. Feature selection improved model interpretation by limiting the number of predictors utilized, picking only those that are clinically relevant, and enables implementing the algorithm online as in-hospital ACS online mortality calculator. Our model chooses 14 predictors that are applicable to both STEMI and NSTEMI patients, eliminating the need for two separate algorithms such as the TIMI score. The variables are simple variables that can be obtained through routine blood tests and clinical examination. In terms of clinical application, the algorithm is deployed as a risk calculator online on the Hospital UITM intranet, which is not accessible to the public due to the study’s ongoing testing, at https://myheartacs.uitm.edu.my. We have developed the algorithm based on previous study on Asian STEMI patients https://myheartstemi.uitm.edu.my/home.php [28].

Asian patients require a population-specific, accurate, and user-friendly algorithm for better resource allocation. To the best of our knowledge, no studies on multiethnic Asian populations using predictive algorithms have been published. We are the first study to do so, and we have successfully implemented the algorithm for clinical use. Given the NCVD registry’s ethnic make-up of Malay, Chinese, and Indian descendants, the study’s generalizability is relevant to Asians in general. It is especially important for Malaysia, Brunei, and Singapore, as well as other Asian countries like China and India [78].

Future research will concentrate on the real-time validation of the best algorithm including several local hospitals for the continual assessment of its reliability. It is possible to improve mortality prediction by using population-specific DL models in conjunction with conventional risk score methods, which can assist clinicians in better allocate limited resources while also improving communication with patients and raising their level of awareness.

### Study limitations

The purpose of this study was to evaluate the performance of a DL-based model for in-hospital mortality to that of a clinical prognostic model for 30-day mortality TIMI. Its robustness might be enhanced if factors were included and compared to other scoring systems, such as GRACE and the Heart Score. This attempt was thwarted by the absence of certain factors. We recognized that missing variables could result in a skewed outcome. We attempted to mitigate this effect by using the same population for both the TIMI and DL-based scores. It is difficult to control selection bias inside registries. We expect that subsequent investigations conducted in the actual world will corroborate our findings. Deep learning with interpretability has been researched and will be our next focus [79, 80]. In contrast to medical expertise, machine learning and deep learning algorithms rely on the relationship between variables. We are concerned that the algorithm created in this study may be biased by the representativeness of the training data. As a result, we constructed and released the algorithm online, along with a repository for future results, as validation of this model in various situations is important.

## Conclusion

In conclusion, we created and tested a new model for ACS risk stratification in Asian patients by incorporating machine learning feature selection with a deep-learning classification algorithm. For ACS patients, the best performing model DL (SVM selected var) predicted in-hospital mortality better than traditional risk scores and other machine-learning approaches. This study determined the viability of the proposed algorithm, which is based on a combination of machine learning and deep learning. Cardiology model that can be used in practice to make precise decisions.

## References

[1] P. B, Jeong YH. Acute coronary syndrome in the Asia-Pacific region. International journal of cardiology. 2016;: 861–869. doi: 10.1016/j.ijcard.2015.04.073 26476044

[2] Hoo FK, Foo YL, Lim SM, Ching SM, Boo YL. Acute coronary syndrome in young adults from a Malaysian tertiary care centre. Pakistan journal of medical sciences. 2016;: 841–845. doi: 10.12669/pjms.324.9689 27648025 PMC5017088

[3] Malaysia DoS. Department of Statistics Malaysia. [Online].; 2020. Available from: www.dosm.gov.my.

[4] Kumar A, Cannon CP. Acute coronary syndromes: diagnosis and management, part I. Mayo Clinic proceedings, 84(10). 2009;: 917–938. doi: 10.1016/S0025-6196(11)60509-0 19797781 PMC2755812

[5] Williams D, Ramgopal R, Gdowski M, Dretler A, Bhat P. Acute Coronary Syndromes, Unstable Angina, and Non–ST-Segment Elevation Myocardial Infarction. Washington Manual of Medical Therapeutics (35th edition), Wolters Kluwer Health. 2016.

[6] Bawamia B, Mehran R, Qiu WL, Vijay K. Risk scores in acute coronary syndrome and percutaneous coronary intervention: A review. American Heart Journal, 165(4). 2013;: 441–450. doi: 10.1016/j.ahj.2012.12.020 23537960

[7] Kwon J, Jeon K, Kim H, Kim M, Lim S, Kim K, et al. Deep-learning-based out-of-hospital cardiac arrest prognostic system to predict clinical outcomes. Resuscitation,139. 2019;: 84–91. doi: 10.1016/j.resuscitation.2019.04.007 30978378

[8] Joshi P, Islam S, Pais P, Reddy S, Dorairaj P, Kazmi K. Risk Factors for Early Myocardial Infarction in South Asians Compared with Individuals in Other Countries. JAMA. 2007;: 297(3), 286. doi: 10.1001/jama.297.3.286 17227980

[9] Mills K, Bundy J, Kelly T, Reed J, Kearney P, Reynolds K. Abstract 16828: Global Disparities of Hypertension Prevalence and Control: A Systematic Analysis of Population-based Studies From 90 Countries. Circulation. 2015;: 132(suppl_3). 10.1161/CIRCULATIONAHA.115.018912 PMC4979614 27502908

[10] Teo BW, Chan GC, Leo CCH, Tay JC, Chia YC, Siddique S, et al. Hypertension and chronic kidney disease in Asian populations. The Journal of Clinical Hypertension, 23(3). 2021;: 475–480. doi: 10.1111/jch.14188 33538081 PMC8029545

[11] Ahmad WAW, Zambahari R, Ismail O, Sinnadurai J, Rosman A, Piaw CS, et al. Malaysian national cardiovascular disease database (NCVD)–acute coronary syndrome (ACS) registry: how are we different? CVD Prevention and Control, 6(3). 2011;: 81–89.

[12] Rana A, de Souza R, Kandasamy S, Lear S, Anand S. Cardiovascular risk among South Asians living in Canada: a systematic review and meta-analysis. CMAJ Open, 2(3). 2014;: E183–E191. doi: 10.9778/cmajo.20130064 25295238 PMC4183167

[13] Ridker PM, Danielson E, Fonseca, H. FA. Rosuvastatin to prevent vascular events in men and women with elevated C-reactive protein. The New England Journal of Medicine Med, 359(21). 2008;: 2195–2207. doi: 10.1056/NEJMoa0807646 18997196

[14] Tang F, Ishwaran H. Random Forest missing data algorithms. Stat Analysis Data Mining, 10(6). 2017;: 63–77. doi: 10.1002/sam.11348 29403567 PMC5796790

[15] Van Buuren S, Groothuis-Oudshoorn K. Mice: Multivariate Imputation by Chained Equations in R. J Stat Softw 45(3). 2011;: 1–67.

[16] Najafabadi MM, Villanustre F, Khoshgoftaar TM, Seliya N, Wald R, Muharemagic E. Deep learning applications and challenges in big data analytics. Journal of big data, 2(1). 2015;: 1–21.

[17] Poh K, Ambegaonkar B, Baxter C, Brudi P, Buddhari W, Chiang F, et al. Low-density lipoprotein cholesterol target attainment in patients with stable or acute coronary diseasee in the Asia-Pacific region: results from the Dyslipidemia International Study II. Eur J Prev Cardiol, 25(18). 2018;: 1950–1963. 30198749 10.1177/2047487318798927

[18] Li YH, Chao TH, Liu PY, Ueng KC, Yeh HI. Lipid Lowering Therapy for Acute Coronary Syndrome and Coronary Artery Disease: Highlights of the 2017 Taiwan Lipid Guidelines for High-Risk Patients. Acta Cardiologica Sinica, 34(5). 2018;: 371–378. doi: 10.6515/ACS.201809_34(5).20180629A 30271086 PMC6160516

[19] Wang Y, Yan B, Nichol M, Tomlinson B, Lee V. Real-world study of low-density lipoprotein cholesterol levels and cardiovascular outcomes in Chinese: A retrospective cohort study in post-percutaneous coronary intervention acute coronary syndrome patients. Int J Cardiol. 2017;: 18–24. doi: 10.1016/j.ijcard.2017.07.016 29121725

[20] Shouval R, Hadanny A, Shlomo N, Iakobishvili Z, Unger R, Zahger D, et al. Machine learning for prediction of 30-day mortality after ST elevation myocardial infraction: An Acute Coronary Syndrome Israeli Survey data mining study. International Journal of Cardiology. 2017;: 7–13. doi: 10.1016/j.ijcard.2017.05.067 28867023

[21] Kasim SS, Malek S, Ibrahim KKS, Aziz MF. Risk stratification of Asian patients after ST-elevation myocardial infarction using machine learning methods. European Heart Journal, 41(2). 2020.

[22] Yang L. Artificial intelligence: a survey on evolution, models, applications and future trends. Journal of Management Analytics, 6(1). 2019;: 1–29.

[23] Yanishi K, Nakamura T, Nakanishi N, Yokota I, Zen K, Yamano T. A Simple Risk Stratification Model for ST-Elevation Myocardial Infarction (STEMI) from the Combination of Blood Examination Variables: Acute Myocardial Infarction-Kyoto Multi-Center Risk Study Group. PLoS One. 2016;: 11.e01166391. doi: 10.1371/journal.pone.0166391 27835698 PMC5105954

[24] Li X, Liu H, Yang J, Xie G, Xu M, Yang Y. Using Machine Learning Models to Predict In-Hospital Mortality for ST-Elevation Myocardial Infarction Patients. Studies in health technology and informatics, 245. 2017;: 476–480. 29295140

[25] Sherazi S, Jeong YJ, Jae MH, Bae JW, Lee JY. A machine learning-based 1-year mortality prediction model after hospital discharge for clinical patients with acute coronary syndrome. Health informatics journal, 26(2). 2020;: 1289–1304. doi: 10.1177/1460458219871780 31566458

[26] Lee W, Lee J, Woo SI, Choi SH, Bae JW, Jung S, et al. Machine learning enhances the performance of short and long-term mortality prediction model in non-ST-segment elevation myocardial infarction. Scientific reports 11. 2021;: 1–14. 34145358 10.1038/s41598-021-92362-1 PMC8213755

[27] Yang J, Li X, Chen T, Li Y, Xie G, Yang Y. Machine learning models to predict in-hospital mortality for ST-elevation myocardial infarction: from china acute myocardial infarction (cami) registry. Journal of the American College of Cardiology, 71(11S). 2018;: A236–A236.

[28] Aziz F, Malek S, Ibrahim KS, Raja Shariff RE, Wan Ahmad WA, Ali RM, et al. Short-and long-term mortality prediction after an acute ST-elevation myocardial infarction (STEMI) in Asians: A machine learning approach. PloS one, 16(8). 2021;: e0254894. doi: 10.1371/journal.pone.0254894 34339432 PMC8328310

[29] Wang C, Zou J, Zhang J, Wang M, Wang R. Feature extraction and recognition of epileptiform activity in EEG by combining PCA with ApEn. Cognitive neurodynamics, 4(3). 2010;: 233–240. doi: 10.1007/s11571-010-9120-2 21886676 PMC2918747

[30] Weberpals J, Becker T, Davies J, Schmich F, Rüttinger D, Theis FJ, et al. Deep learning-based propensity scores for confounding control in comparative effectiveness research: A large-scale, real-world data study. Epidemiology, 32(3). 2021;: 378–388. doi: 10.1097/EDE.0000000000001338 33591049

[31] Le CY, Bengio Y, Hinton G. Deep learning. Nature 521. 2015;: 436–444. doi: 10.1038/nature14539 26017442

[32] Motwani M, Dey D, Berman DS, Germano G, Achenbach S, Al-Mallah MH, et al. Machine learning for prediction of all-cause mortality in patients with suspected coronary artery disease: a 5-year multicentre prospective registry analysis. European Heart Journal. 2017;: 38(7). doi: 10.1093/eurheartj/ehw188 27252451 PMC5897836

[33] Chen YH, Huang SS, Lin SJ. TIMI and GRACE Risk Scores Predict Both Short-Term and Long-Term Outcomes in Chinese Patients with Acute Myocardial Infarction. Acta Cardiologica Sinica, 34(1). 2018;: 4–12. doi: 10.6515/ACS.201801_34(1).20170730B 29375219 PMC5777938

[34] Kasim S, Malek S, Ibrahim K, Amir P, Aziz M. Investigating performance of deep learning and machine learning risk stratification of Asian in-hospital patients after ST-elevation myocardial infarction. European Heart Journal-Digital Health, 2(4). 2021;: ztab104-3068.

[35] Ahmad W, Zambahari R, Ismail O, Sinnadurai J, Rosman A, Piaw C, et al. Malaysian national cardiovascular disease database (NCVD)–acute coronary syndrome (ACS) registry: how are we different. CVD Prevention and Control 6(3). 2011;: 81–89.

[36] Wan K, Zhao J, Huang H, Zhang Q, Chen X, Zeng Z, et al. The association between triglyceride/high-density lipoprotein cholesterol ratio and all-cause mortality in acute coronary syndrome after coronary revascularization. PloS one, 10(4). 2015;: e0123521. doi: 10.1371/journal.pone.0123521 25880982 PMC4399840

[37] Peng Y, Du X, Rogers K, Wu Y, Gao R, Patel A. Predicting In-Hospital Mortality in Patients with Acute Coronary Syndrome in China. The American Journal of Cardiology, 2017 October; 120(7). 2017;: 1077–83. doi: 10.1016/j.amjcard.2017.06.044 28818316

[38] Wallert J, Tomasoni M, Madison G, Held C. Predicting two-year survival versus nonsurvival after first myocardial infarction using machine learning and Swedish national register data. BMC medical informatics and decision making. 2017;: 99. doi: 10.1186/s12911-017-0500-y 28679442 PMC5499032

[39] Stekhoven D, Buhlmann P. MissForest—non-parametric missing value imputation for mixed type data. Bioinformatics 28(1). 2012;: 112–8. doi: 10.1093/bioinformatics/btr597 22039212

[40] Buuren S, Groothuis-Oudshoorn K. mice: Multivariate imputation by chained equations in R. Journal of statistical software. 2010;: 1–68.

[41] Kuhn M, Johnson K. Applied predictive modeling. Springer. 2013.

[42] Dalwinder S, Birmohan S. Investigating the impact of data normalization on classification performance. Applied Soft Computing. 2020;: 97.

[43] Ioffe S, Szegedy C. Batch Normalization: Accelerating Deep Network Training by Reducing Internal Covariate Shift. 2015.

[44] Jason B. Train Faster, Reduce Overfitting, and Make Better Prediction. Better Deep Learning. 2019.

[45] Fawcett T. An introduction to ROC analysis. Pattern recognition letters 27, no. 8. 2006;: 861–874.

[46] Trevethan R. Sensitivity, specificity, and predictive values: foundations, pliabilities, and pitfalls in research and practice. Frontiers in public health, 5. 2017;: 307. doi: 10.3389/fpubh.2017.00307 29209603 PMC5701930

[47] Sun XZ, Yang Z. Generalized McNemar’s Test for Homogeneity of the Marginal Distributions. SAS Global Forum, Statistics and Data Analysis. 2008;: 382.

[48] Omolola AA, Wilella DB. Analysis of Paired Dichotomous Data: A Gentle Introduction to the McNemar Test in SPSS. Journal of MultiDisciplinary Evaluation, 8(17). 2012.

[49] Bengio Y, Nadeau C. Inference for the Generalization Error. CIRANO. 1999.

[50] Miao JY, Niu LF. A survey on Feature Selection. Procedia Computer Science 91. 2020;: 919–926.

[51] Genuer R, Poggi J, Tuleau-Malot C. Variable selection using random forests Pattern Recognition Letters. J. PATREC. 2010;: 14.

[52] Marcílio WE, Eler DM. From explanations to feature selection: assessing shap values as feature selection mechanism. Ieee. 2020;: 340–347.

[53] Rozemberczki B, Watson L, Bayer P, Yang HT, Kiss O, Nilsson S, et al. The Shapley Value in Machine Learning. arXiv preprint. 2022;: 2202.05594.

[54] Correia LC GG, Kalil F, Ferreira F, Carvalhal M, Oliveira R, Silva A, et al. Prognostic value of TIMI score versus GRACE score in ST-segment elevation myocardial infarction. Arquivos brasileiros de cardiologia. 2014; 103: 98–106. doi: 10.5935/abc.20140095 25029471 PMC4150660

[55] Pencina M, D’Agostino R Sr, D’Agostino R Jr, Vasan R. Evaluating the added predictive ability of a new marker: from area under the ROC curve to reclassification and beyond. Statistics in medicine, 27(2). 2008;: 157–72. doi: 10.1002/sim.2929 17569110

[56] Dietterich T. Approximate statistical tests for comparing supervised classification learning algorithms. Neural computation, 10(7). 1998;: 1895–1923. doi: 10.1162/089976698300017197 9744903

[57] Raschka S. Model evaluation, model selection, and algorithm selection in machine learning. arXiv preprint. 2018;: arXiv:1811.12808.

[58] Team RDC. R: A language and environment for statistical computing. Vienna: R Foundation for Statistical Computing, Vienna, Austria.; 2011.

[59] Lee G, Kim SM, Choi S, Kim K, Jeong SM, Son JS, et al. The effect of change in fasting glucose on the risk of myocardial infarction, stroke, and all-cause mortality: a nationwide cohort study. Cardiovascular diabetology. 2018; 17(1): 1–10. 29626936 10.1186/s12933-018-0694-z PMC5889526

[60] Ahmad W, Ali R, Khanom M, Han C, Bang L, Yip A, et al. The journey of Malaysian NCVD—PCI (National Cardiovascular Disease Database—Percutaneous Coronary Intervention) Registry: A summary of three years report. International of Journal of Cardiology. 2013;: 161–164. 10.1016/j.ijcard.2011.08.015 21920614

[61] Kuhn M, Johnson K. Classification trees and rule-based models. In Applied predictive modeling. New York: Springer; 2013. p. 369–413.

[62] Aziida N, Malek S, Aziz F, Ibrahim KS, Kasim S. Predicting 30-Day Mortality after an Acute Coronary Syndrome (ACS) using Machine Learning Methods for Feature Selection, Classification and Visualisation. Sains Malaysiana. 2021; 50(3): 753–768.

[63] Chimparlee N, Chaipromprasit J, Athisakul S, Lertsuwunseri V, Buddhari W, Udayachalerm W, et al. Comparison Between Timi and Grace Scores as A Predictor for Short-And Long-Term Outcome in Patients with Acute St-Elevation Myocardial Infarction. Journal of the American College of Cardiology. 2018.

[64] Timbol A, Villaluna R, Punzalan F. 106: Timi Risk Score For Stemi A Validation Study Among Filipinos For Predicting In-Hospital Mortality. Critical care medicine. 2015;: 28.

[65] González-Pacheco H, Alexandra AM, Amada ÁS, Úrsulo JH, Félix D, Guering EL, et al. The TIMI risk score for STEMI predicts in-hospital mortality and adverse events in patients without cardiogenic shock undergoing primary angioplasty. Arch Cardiol Mex 82, no. 1. 2012;: 7–13. 22452860

[66] Morrow DA, Antman EM, Charlesworth A, Cairns R, Murphy SA, de Lemos JA, et al. TIMI risk score for ST-elevation myocardial infarction: a convenient, bedside, clinical score for risk assessment at presentation: an intravenous nPA for treatment of infarcting myocardium early II trial substudy. Circulation. 2000; 102(17): 2031–2037. doi: 10.1161/01.cir.102.17.2031 11044416

[67] Feder SL, Schulman-Green D, Geda M, Williams K, Dodson JA, Nanna MG, et al. Physicians’ perceptions of the Thrombolysis in Myocardial Infarction (TIMI) risk score in older adults with acute myocardial infarction. Heart & lung: the journal of critical care. 2015;: 376–381. doi: 10.1016/j.hrtlng.2015.05.005 26164651 PMC4567390

[68] Selvarajah S, Fong A, Selvaraj G, Haniff J, Hairi N, Bulgiba A, et al. Impact of cardiac care variation on ST-elevation myocardial infarction outcomes in Malaysia. The American journal of cardiology. 2013;: 1270–6. doi: 10.1016/j.amjcard.2013.01.271 23415636

[69] Krishna GA, Umesh UT, Eva KL, Li J, Keith A. Does simplicity compromise accuracy in ACS risk prediction? A retrospective analysis of the TIMI and GRACE risk scores. PLoS ONE. 2009. 10.1371/journal.pone.0007947 PMC2776353 19956773

[70] Cheng JM, Helming AM, van Vark LC, Kardys, Den Uil CA, Jewbali LS, et al. A simple risk chart for initial risk assessment of 30-day mortality in patients with cardiogenic shock from ST-elevation myocardial infarction. European Heart Journal: Acute Cardiovascular Care. 2016; 5(2): 101–107. doi: 10.1177/2048872615568966 25589634

[71] Tang EW, Wong CK, Herbison P. Global Registry of Acute Coronary Events (GRACE) hospital discharge risk score accurately predicts long-term mortality post acute coronary syndrome. American heart journal, 153(1). 2007;: 29–35. doi: 10.1016/j.ahj.2006.10.004 17174633

[72] Van Den Berg P, Body R. The HEART score for early rule out of acute coronary syndromes in the emergency department: a systematic review and meta-analysis. European Heart Journal: Acute Cardiovascular Care, 7(2). 2018;: 111–119. doi: 10.1177/2048872617710788 28534694

[73] Granger C, Goldberg R, Dabbous O, Pieper K, Eagle K, Cannon C, et al. Predictors of hospital mortality in the global registry of acute coronary events. Archives of internal medicine. 2003 October; 163(19): 2345–2353. doi: 10.1001/archinte.163.19.2345 14581255

[74] Venkatason P, Zubairi Y, Ahmad W, Hafidz M, Ismail M, Hadi M, et al. Inhospital mortality of cardiogenic shock complicating ST-elevation myocardial infarction in Malaysia: a retrospective analysis of the Malaysian National Cardiovascular Database (NCVD) registry. BMJ open. 2019. doi: 10.1136/bmjopen-2018-025734 31061031 PMC6502239

[75] Zuhdi ASM, Ahmad WAW, Zaki RA, Mariapun J, Ali RM, Sari NM, et al. Acute coronary syndrome in the elderly: the Malaysian National Cardiovascular Disease Database-Acute Coronary Syndrome registry. Singapore medical journal. 2016; 57(4): 191. doi: 10.11622/smedj.2015145 26768171 PMC4853486

[76] Wan Ahmad W, Sim K. Annual report of the NCVD-ACS Registry, 2014–2015. Kuala Lumpur: National Cardiovascular Disease Database; 2015.

[77] Mao K. Orthogonal forward selection and backward elimination algorithms for feature subset selection. IEEE Transactions on Systems, Man, and Cybernetics, Part B (Cybernetics). 2004 January; 34(1): 629–634. doi: 10.1109/tsmcb.2002.804363 15369099

[78] Irawati S, Wasir R, Floriaan Schmidt A, Islam A, Feenstra T, Buskens E, et al. Long-term incidence and risk factors of cardiovascular events in Asian populations: systematic review and meta-analysis of population-based cohort studies. Current Medical Research and Opinion, 35(2). 2019;: 291–299. doi: 10.1080/03007995.2018.1491149 29920124

[79] Laufer‐Perl M, Shacham Y, Letourneau‐Shesaf S, Priesler O, Keren G, Roth A, et al. Gender‐ Related Mortality and In‐Hospital Complications Following ST‐Segment Elevation Myocardial Infarction: Data From a Primary Percutaneous Coronary Intervention Cohort. Clinical cardiology. 2015;: 145–149. doi: 10.1002/clc.22363 25728563 PMC6711103

[80] Rajadurai J, Zambahar R, Abdul Rahman AR, Suhaimi. Clinical Practices and Guidelines on Primary & Secondary Prevention of Cardiovascular Disease 2017 Putrajaya, WP Putrajaya: CPG Secretariat; 2017.

